# Role of Folic Acid in the Therapeutic Action of Nanostructured Porous Silica Functionalized with Organotin(IV) Compounds against Different Cancer Cell Lines

**DOI:** 10.3390/pharmaceutics12060512

**Published:** 2020-06-03

**Authors:** Diana Díaz-García, Karla Montalbán-Hernández, Irene Mena-Palomo, Patriciu Achimas-Cadariu, Antonio Rodríguez-Diéguez, Eduardo López-Collazo, Sanjiv Prashar, Karina Ovejero Paredes, Marco Filice, Eva Fischer-Fodor, Santiago Gómez-Ruiz

**Affiliations:** 1 COMET-NANO Group, Departamento de Biología y Geología, Física y Química Inorgánica, ESCET, Universidad Rey Juan Carlos, 28933 Móstoles, Spain; diana.diaz@urjc.es (D.D.-G.); karlamarina.hernandez@gmail.com (K.M.-H.); irene.mena@urjc.es (I.M.-P.); sanjiv.prashar@urjc.es (S.P.); 2 Tumour Biology Department, the Institute of Oncology “Prof. Dr. I. Chiricuta”, RO-400015 Cluj-Napoca, Romania; 3 Innate Immunity Group, Laboratory of Tumour Immunology, IdiPAZ Institute for Health Research, La Paz University Hospital, 28046 Madrid, Spain; elopezc@salud.madrid.org; 4 Department of Surgery, the Institute of Oncology “Prof. Dr. I. Chiricuta”, RO-400015 Cluj-Napoca, Romania; pachimas@umfcluj.ro; 5 Department of Surgery and Gynecological Oncology, the University of Medicine and Pharmacy “Iuliu Hatieganu”, RO-400337 Cluj-Napoca, Romania; 6 Departamento de Química Inorgánica, Universidad de Granada, Facultad de Ciencias, Campus de Fuentenueva, Avda. Fuentenueva s/n, E-18071 Granada, Spain; antonio5@ugr.es; 7 Nanobiotechnology for Life Sciences Group, Department of Chemistry in Pharmaceutical Sciences, Faculty of Pharmacy, Universidad Complutense de Madrid (UCM), Plaza Ramón y Cajal s/n, E-28040 Madrid, Spain; kovejero@ucm.es (K.O.P.); mfilice@ucm.es (M.F.); 8 Microscopy and Dynamic Imaging Unit, Fundación Centro Nacional de Investigaciones Cardiovasculares Carlos III (CNIC), Calle Melchor Fernandez Almagro 3, E-28029 Madrid, Spain; 9 Medfuture-Research Center for Advanced Medicine, the University of Medicine and Pharmacy “Iuliu Hatieganu”, RO-400337 Cluj-Napoca, Romania

**Keywords:** tin, anticancer, mesoporous silica, folic acid, cytotoxicity, FOLR1

## Abstract

The synthesis, characterization and cytotoxic activity against different cancer cell lines of various mesoporous silica-based materials containing folate targeting moieties and a cytotoxic fragment based on a triphenyltin(IV) derivative have been studied. Two different mesoporous nanostructured silica systems have been used: firstly, micronic silica particles of the MSU-2 type and, secondly, mesoporous silica nanoparticles (MSNs) of about 80 nm. Both series of materials have been characterized by different methods, such as powder X-ray diffraction, X-ray fluorescence, absorption spectroscopy and microscopy. In addition, these systems have been tested against four different cancer cell lines, namely, OVCAR-3, DLD-1, A2780 and A431, in order to observe if the size of the silica-based systems and the quantity of incorporated folic acid influence their cytotoxic action. The results show that the materials are more active when the quantity of folic acid is higher, especially in those cells that overexpress folate receptors such as OVCAR-3 and DLD-1. In addition, the study of the potential modulation of the soluble folate receptor alpha (FOLR1) by treatment with the synthesized materials has been carried out using OVCAR-3, DLD-1, A2780 and A431 tumour cell lines. The results show that a relatively high concentration of folic acid functionalization of the nanostructured silica together with the incorporation of the cytotoxic tin fragment leads to an increase in the quantity of the soluble FOLR1 secreted by the tumour cells. In addition, the studies reported here show that this increase of the soluble FOLR1 occurs presumably by cutting the glycosyl-phosphatidylinositol anchor of membrane FR-α and by the release of intracellular FR-α. This study validates the potential use of a combination of mesoporous silica materials co-functionalized with folate targeting molecules and an organotin(IV) drug as a strategy for the therapeutic treatment of several cancer cells overexpressing folate receptors.

## 1. Introduction

Cancer is one of the most extended diseases in humans leading to about 9.5 million deaths in 2018 [1]. More importantly, in 2018 around 18 million new cancer cases were diagnosed worldwide, and it is expected that in 2040 this will rise to about 30 million, which would make it the illness with the highest incidence in the population ahead of cardiovascular diseases [2]. When one analyses the most common types of cancers worldwide, lung, female breast, bowel and prostate cancer are at the top of the list and constitute around 40% of the worldwide total number of cancer cases [1]. Due to the devastating number of cases and deaths caused by cancer, some of the most extended efforts of the scientific community are presently focused on finding novel treatments or improving traditional drugs by novel formulations, which may lead to potential alternatives for preclinical or clinical trials.

The current therapies for the treatment of cancer are focused on personalized treatments [3,4], radiotherapy [5], hormonotherapy [6], chemotherapy and some other potential biological therapies [7]. However, when chemotherapeutic treatment of cancer is considered, platinum-based metal drugs [8,9] have been, and still are, the most extended drugs to help in controlling the exponential growth of cancer cells in several cancers [10]. Nevertheless, the use of platinum metallodrugs is limited due to their toxicity, low stability and speciation in physiologic media and some additional problems associated with poor tumour-targeting strategies [11]. The search to overcome these problems has led to intensive research of other metal-based drugs [10,12], metallodrug-functionalized systems with controlled drug loading, and a targeted and selective drug release, which improves their potential applicability in humans [11,13,14].

In this context, since 2009 our team has been working on the use of porous silica-based nanostructured materials such as MCM-41, SBA-15, hexagonal mesoporous silica (HMS), MSU-2, KIT-6 or mesoporous silica nanoparticles (MSNs) functionalized with different metallodrugs [15,16,17,18,19,20,21,22,23,24,25,26,27]. Previous studies have shown that these systems usually work as “non-classical” drug delivery systems, namely, acting as an entire nanoparticulated therapeutic system, without the need of the released metallodrug to be cytotoxic. Additional work of other groups has also pointed to an inexistent or very low release of the metal-containing cytotoxic species to the physiologic medium [28,29,30,31,32].

Considering that most of the studied systems do not bear any potential targeting molecule to detect and potentiate the active and selective transport of the therapeutic species to the cancer cell line, an enhanced permeability and retention (EPR) effect was considered to be responsible for their slight selectivity towards cancer cell lines (in comparison with normal cell lines) [15,16,17,18,19,20,21,22,23,24,25,26,27,28,29,30,31,32]. Nevertheless, very recent reports have studied the possibility of including a folate fragment for a more effective targeting of cancer cells, which has been partially achieved both in vitro [33] and in vivo [34] when using organotin(IV)-functionalized mesoporous silica nanoparticles. Using this strategy of introducing folate fragments, an increase in the selectivity of functionalized-nanostructured silica systems has been achieved using a wide variety of organic approved drugs; however, the biological mechanism of action, including the receptor study of these systems, has still not been elucidated [35,36,37,38,39,40,41,42,43,44,45,46,47].

As the number of reports of folate-containing metallodrug-functionalized mesoporous silica nanomaterials is very low and limited to the previous studies of our team with tin(IV) derivatives [33,34] and other therapeutic gold(III) compounds [48], a detailed study was proposed to determine some biological aspects related to the use of these kinds of fragments in cancer chemotherapy in vitro. In general, the folate receptor is a recognised biomarker for tumour cells, usually enabling improvement in cancer cell uptake and potential delivery of the drugs inside the cells [49]. It is well known that human tumour cells are characterized by overexpression of the folate receptor-α (FR-α) on their membrane, FR-α also being evidenced in the cytoplasm of the tumour cells [50]. Folate receptor-α is anchored to the surface of the cell by a glycosyl-phosphatidylinositol molecule, and it is also present inside the cell [51,52] and facilitates the uptake into the living cells of folic acid or B9 vitamin, which is essential for cellular growth and replication. Most of the normal tissues lack expression of FOLR1, but they are overexpressed on epithelial malignant cells, mostly in solid tumours such as ovarian tumours [53], breast cancer, cervical cancer, non-small cell lung adenocarcinoma and colorectal cancer. In tumour cells, folic acid activates the signal transducer and activator of transcription 3 (STAT3) pathway through FR-α [54]. Knowing that folic acid receptors play an important role in carcinogenesis, FR-α was quickly identified as an attractive therapeutic target. The abundance of folate receptor in tumour tissues and its ability to internalize folate acid derivatives has been used as a promising drug delivery target to develop folic acid conjugated drugs.

The soluble form of the folate binding protein or FOLR1 is present in the human blood serum [55]. Studies suggest that the folate binding protein is produced by tumour cells rich in FR-α and is released into the bloodstream by tumours [56]. The level of soluble FOLR1 is selectively higher in cancer patients [57], a prominent example being ovarian carcinoma, where FOLR1 is upregulated. Therefore, this protein can act as a sensitive tumour marker. In ovarian carcinoma cells, FOLR1 was intensely studied, and it was confirmed that the folate receptor is abundant in the OVCAR-3 cell line [58]; in A2780 cells, the folate receptor positive expression was confirmed [59], but the studies indicated a lower expression than in other malignant ovary cell lines. The overexpression of FOLR1 or FR-α was previously confirmed on colon DLD-1 [60], while in the epidermal squamous carcinoma A431 tumour cell line FOLR1 expression was reduced [61].

Herein, the potential cytotoxic action against cells overexpressing folate receptors has been studied. In addition, potential modulation of the soluble folate receptor alpha (FOLR1) by treatment with simple organotin(IV) functionalized mesoporous nanostructured silica systems based on micronic silica particles (MSU-2) and mesoporous silica nanoparticles (MSNs) of about 80 nm has been tested. This study has been carried out in order to observe if the size of the silica-based systems influences both the cytotoxic and potential folate-targeting actions. Biological tests with tin-functionalized silica materials have been carried out using OVCAR-3, DLD-1, A2780 and A431 tumour cell lines, showing that functionalization of the nanostructured silica with a triphenyltin(IV) moiety group, together with a relatively high concentration of folic acid, leads to an increase in the quantity of soluble FOLR1 secreted by tumour cells. In addition, the combination of folic acid in high quantity and the organotin(IV) cytotoxic fragment induces a high cytotoxic activity against cells overexpressing folate receptors. Furthermore, the studies reported here show that an increase in the quantity of soluble FOLR1 occurs presumably by cutting the glycosyl-phosphatidylinositol anchor of membrane FR-α and by the release of intracellular FR-α.

## 2. Materials and Methods

### 2.1. General Remarks on the Synthesis and Characterization of the Materials

All synthesis and functionalization reactions were performed using standard Schlenk tube techniques in an atmosphere of dry nitrogen, except those of the synthesis of the silica materials MSU-2 and MSN and the coupling reactions to incorporate the folate fragment, which were directly carried out in air. All the solvents of the synthetic part were distilled from the appropriate drying agents and degassed before use. The reagents used in the preparation of the starting materials MSU-2 and MSN, namely hexadecyltrimethylammonium bromide 99 + % (CTAB, MW = 364.456 g/mol, Acros Organics, Geel, Belgium), sodium hydroxide granulated (NaOH, MW = 40 g/mol, Scharlau, Barcelona, Spain), tetraethyl orthosilicate 98% (TEOS, MW = 208.33 g/mol, Sigma Aldrich, Tres Cantos, Spain), NaF ≥ 99% (MW = 41.99 g/mol, Sigma Aldrich, Tres Cantos, Spain) and Tergitol 70% in H_2_O (MW = 1980 g/mol, Sigma Aldrich, Tres Cantos, Spain), were used as received without further purification. The reagents used for the functionalization reactions, namely 3-aminopropyltriethoxysilane 99% (AP, MW = 319.426 g/mol, Sigma Aldrich, Tres Cantos, Spain), 3-mercaptopropyltriethoxysilane 94% (MP, MW = 238.42 g/mol, Sigma Aldrich, Tres Cantos, Spain), triphenyltin(IV) chloride 95% (MW = 385.47 g/mol, Sigma Aldrich, Tres Cantos, Spain), triethylamine ≥ 99% (MW = 101.19 g/mol, Sigma Aldrich, Tres Cantos, Spain), MES monohydrate ≥ 99% (MW = 213.25 g/mol, Sigma Aldrich, Tres Cantos, Spain), *N*-(3-dimethylaminopropyl)-*N*’-ethylcarbodiimide ≥ 97% (EDAC, MW = 155.24 g/mol, Sigma Aldrich, Tres Cantos, Spain), *n*-hydroxysuccinamide 98% (NHS, MW = 115.09 g/mol, Sigma Aldrich, Tres Cantos, Spain), 2-mercaptoethanol ≥ 99% (MW = 78.13 g/mol, Sigma Aldrich, Tres Cantos, Spain), hydroxylamine hydrochloride 99% (MW = 69.49 g/mol, Sigma Aldrich, Tres Cantos, Spain) and folic acid ≥ 97% (FA, MW = 441.4 g/mol, Sigma Aldrich, Tres Cantos, Spain), were all used directly without further purification.

^13^C-CP MAS spectra were recorded on a Varian-Infinity Plus Spectrometer at 400 MHz operating at 100.52 MHz proton frequency (4 μs 90° pulse, 4000 transients, spinning speed of 6 MHz, contact time 3 ms, pulse delay 1.5 s). X-ray diffraction (XRD) patterns of the systems were obtained on a Philips Diffractometer model PW3040/00 X’Pert MPD/MRD at 45 kV and 40 mA, using a wavelength Cu Kα (λ = 1.5418 Å). Sn wt% determination by X-ray fluorescence was carried out with an X-ray fluorescence spectrophotometer Philips MagiX with an X-ray source of 1 kW and a Rh anode using a helium atmosphere. FT-IR analysis was determined with a Termo Nicolet Avatar 380 FT-IR spectrometer with a Michelson type filter interferometer N_2_ gas adsorption–desorption isotherms performed using a Micromeritics ASAP 2020 analyzer. Thermogravimetry analyses were obtained on a Shimadzu mod. DSC-50Q (Shimadzu, Kioto, Japan) operating between 30 and 800 °C (ramp 20 °C/min) at an intensity of 50 A.

Scanning electron micrographs and morphological analyses were carried out on a XL30 ESEM Philips with an energy-dispersive spectrometry system (EDS) or with a high-resolution FEG-SEM Nova Nano SEM230. In all cases for SEM measurements, the samples were treated with a sputtering method with the following parameters: sputter time 100 s, sputter current 30 mA and film thickness 20 nm using a sputter coater BAL-TEC SCD 005. The metal used for the sputtering method was gold. Conventional transmission electron microscopy (TEM) was carried out on a TECNAI 20 Philips, operating at 200 kV and using copper grids. Dynamic light scattering (DLS) measurements were carried out on a Zetasizer Nano Zen 3600 (Malvern, UK), diluting samples in potassium nitrate (KNO_3_, 10^−2^ M).

### 2.2. Synthesis of Mesoporous Silica Nanoparticles (MSNs)

The synthesis of MSNs was carried out with a slight modification of the experimental procedure reported by Zhao et al. [62]. An aqueous solution of CTAB (1.00 g, 2.74 mmol) was prepared in 480 mL of Milli-Q water. Sodium hydroxide (2 M, 3.5 mL) was then added to the solution and the temperature increased to 80 °C. Afterwards, the silica precursor TEOS (5 mL, 22.4 mmol) was added dropwise under vigorous stirring, and the mixture was allowed to react for an additional 2 h. The white precipitate was isolated by filtration, washed with abundant Milli-Q water and with methanol (2 × 20 mL) and dried for 24 h at 80 °C in a stove. Finally, a calcination process at 500 °C was carried out for 24 h with an increasing temperature ramp of 1 °C/min.

### 2.3. Synthesis of Porous Micronic Silica Particles of the MSU-2 Type (MSU-2)

The synthesis of MSU-2 was carried out with a slight modification of the experimental procedure reported by Pérez-Quintanilla et al. [63]. To summarize, 1562.5 mL of nanopure (Milli-Q) water was placed in a 2 L flask at 35 °C. Subsequently, 78.1 g of Tergitol was added to the nanopure water and the mixture stirred. After completely dissolving Tergitol, 52.0 g of TEOS was added dropwise to the mixture. The solution was stirred for 30 min, the stirring was stopped, and the solution was left under static ageing for 20 h at 35 °C. The solution slowly turned into a white suspension, stirring was set to 800 rpm, and 26 mL of NaF 0.24 M was added dropwise. The temperature was increased to 55 °C, and the solution was left stirring for 48 h. The reaction was then stopped, and the solid product was isolated by filtration and thoroughly washed with nanopure water to ensure as much surfactant as possible was removed. The product was dried overnight in the oven. The dry product was finally calcined at 650 °C for 24 h, with a temperature ramp of 1 °C/min.

### 2.4. Functionalization of Silica Materials with Amino Ligand—Synthesis of MSN-AP or MSU-2-AP

The functionalization of MSN was carried out using similar procedures described previously [64,65]. To summarize, MSN or MSU-2 (1.00 g) was partially dehydrated under vacuum at 80 °C for 24 h and suspended in 20 mL of dry toluene. The mixture was then treated with a solution of 3-aminopropyltriethoxysilane (AP) (2.00 mL, 9.01 mmol) in 30 mL of dry toluene. The mixture was stirred for 48 h at 110 °C, and then filtered to isolate the precipitate, which was washed with toluene and diethylether and subsequently dried for 24 h at 80 °C.

### 2.5. Incorporation of Folate Fragment—Synthesis of MSN-AP-FA or MSU-2-AP-FA

For the functionalization of MSN-AP or MSU-2-AP with folic acid, an EDAC coupling reaction was carried out. For this, 50 mL 0.1 M of MES Buffer was prepared with 0.5 M sodium chloride. Subsequently, 50 mg of folic acid (10% functionalization of MSN-AP or MSU-2-AP) was dissolved in 12.5 mL of DMSO (in an ultrasound bath), and this solution was added to the MES buffer solution containing 20 mg (0.104 mmol) of EDAC and 30 mg (0.261 mmol) of NHS. The mixture was allowed to react under vigorous stirring for 15 min at room temperature. Subsequently, 0.5 g of MSN-AP or MSU-2-AP was added to the EDAC solution and allowed to react for 2 h, stirring at room temperature. A total of 7 μL (0.107 mmol) of 2-mercaptoethanol was then added to the solution, and the mixture was stirred for an additional 30 min. Finally, 41.5 mg (0.597 mmol) of hydroxylamine was added to quench the reaction. The mixture was then centrifuged (6000 rpm, 10 min), and the isolated solid product was washed with DMSO, ethanol, water and diethylether (1 × 10 mL each). The product was dried in a stove at 80 °C overnight.

### 2.6. Incorporation of a High Quantity of Folate Fragment—Synthesis of MSN-AP-FA25 or MSU-2-AP-FA25

For the functionalization of MSN-AP or MSU-2-AP with 25% of folic acid, an EDAC coupling reaction was carried out using an identical procedure to that described for the preparation of MSN-AP-FA or MSU-2-AP-FA but using a higher amount of folic acid. The quantities used were 50 mL 0.1 M of MES buffer prepared with 0.5 M sodium chloride, 125 mg of folic acid (25% functionalization of MSN-AP or MSU-2-AP) dissolved in 25 mL of DMSO, MES buffer solution containing 50 mg (0.260 mmol) of EDAC and 75 mg (0.652 mmol) of NHS. 0.5 g of MSN-AP or MSU-2-AP, 17.5 μL (0.268 mmol) of 2-mercaptoethanol and 103.8 mg (1.492 mmol) of hydroxylamine.

### 2.7. Incorporation of the Cytotoxic Fragment SnPh_3_—Synthesis of MSN-Sn or MSU-2-Sn

The first step was the preparation of the tin compound Ph_3_Sn{SCH_2_CH_2_CH_2_Si(OEt)_3_}, which was synthesized by the reaction of triphenyltin(IV) chloride and 3-mercaptopropyltriethoxysilane in a Schlenk tube. SnPh_3_Cl (135.0 mg, 0.35 mmol) was dissolved in dry toluene, and subsequently 3-mercaptopropyltriethoxysilane (82.2 μL, 0.35 mmol) and triethylamine (99.4 μL, 0.70 mmol) were added (molar ratio 1:1:2). The suspension was stirred for 24 h at 80 °C. Afterwards, the mixture was filtered, and the filtrate (Ph_3_Sn{SCH_2_CH_2_CH_2_Si(OEt)_3_}) was added to a suspension of 0.25 g of the silica material MSN or MSU-2 (to give a proportion 15% wt Sn/SiO_2_) in 50 mL of toluene. The reaction mixture was stirred at 85 °C for 48 h, and the mixture was then filtered. The isolated solid was then washed with toluene (2 × 10 mL), water (2 × 10 mL) and diethyl ether (2 × 10 mL) and dried overnight at 50 °C under vacuum.

### 2.8. Incorporation of the Cytotoxic Fragment SnPh_3_—Synthesis of MSN-AP-FA-Sn, MSN-AP-FA25-Sn, MSU-2-AP-FA-Sn and MSU-2-AP-FA25-Sn

Incorporation of the cytotoxic tin fragment was carried out in an identical manner to that described previously for the synthesis of MSN-Sn or MSU-2-Sn but using 0.25 g of MSN-AP-FA, MSN-AP-FA25, MSU-2-AP-FA or MSU-2-AP-FA25.

### 2.9. Cell Growth Inhibition

In vitro biologic testing was performed on four human cancer cell lines and a normal cell line. A2780 ovarian carcinoma, DLD-1 colon carcinoma and A431 epidermoid squamous carcinoma cell lines were acquired from Collection of Authenticated Cell Cultures (ECACC, Salisbury, UK); the OVCAR-3 cell line was from American Type Culture Collection (ATCC, Manasses, VA, USA, acquired through LGC Standards GmbH, Wesel, Germany), and the HaCaT immortalized normal keratinocyte cell line was from the Cell Line Service of the German Cancer Research Centre (Heidelberg, Germany).

The A2780 and DLD-1 cells were maintained in RPMI-1640 cell culture media, supplemented with 10% heat-inactivated foetal bovine serum (FBS), 2 mM glutamine, 50 UI/mL penicillin, and 50 mg/mL streptomycin; OVCAR-3 was maintained in RPMI-1640-based media, as described above, with 20% FBS and 0.01 mg/mL insulin from porcine pancreas. A431 was cultivated in Minimum Eagles’ Medium (MEM) supplemented with 1% non-essential amino acids solution, 10% FBS, 2 mM glutamine, 50 UI/mL penicillin, and 50 mg/mL streptomycin. The HaCaT cells were cultivated in Dulbecco’s Modified Eagles’ Medium (DMEM) with 4500 mg/mL glucose, supplemented with 10% FCS, 2 mM glutamine, 50 UI/mL penicillin, and 50 mg/mL streptomycin (all media and supplements were from Sigma-Aldrich Chemie GmbH, Taufkirchen, Germany).

The compounds MSU-2, MSU-2-AP, MSU-2-AP-FA, MSU-2-AP-FA25 and MSU-2-AP-FA25-Sn were dispersed in dimethyl sulfoxide (DMSO from Merck, Darmstadt, Germany) to obtain a stock suspension of 10 mg/mL. The tin concentration incorporated in the various mesoporous structures was different: MSU-2-AP-FA25-Sn contains 5.9% Sn, MSU-2-AP-FA-Sn contains 6.1% Sn, while MSU-2-Sn has 14.1% Sn. An adjustment was made to obtain the same Sn level in all materials. For the cytotoxicity testing, 9.6 mg/mL MSU-2-AP-FA-Sn and 4.25 mg/mL MSU-2-Sn stock solutions were prepared. From all stock solutions, seven serial dilutions were prepared in Phosphate Buffered Saline Solution (PBS, from Sigma Aldrich Chemie GmbH, Taufkirchen, Germany).

The MSN-based materials were also dispersed in DMSO to obtain 10 mg/mL stock solutions for MSN, MSN-AP, MSN-AP-FA, MSN-AP-FA-Sn, MSN-AP-FA25, MSN-AP-FA25-Sn and MSN-Sn. In MSN-AP series, MSN-AP-FA-Sn contained 7.60% Sn, MSN-AP-FA25-Sn 5.70% Sn, while MSN-Sn had 13.90% Sn. To normalize the Sn content, 7.5 mg/mL MSN-AP-FA-Sn and 4.78 mg/mL MSN-Sn solutions were prepared, and these stock solutions were the starting points for the serial dilutions. From each compound, serial dilutions were prepared in PBS.

For the cytotoxicity test, all experiments were carried out under sterile conditions, and the biologic systems were handled in class II biological safety cabinets (ESCO Micro Pte. Ltd., Singapore). The cells were seeded on 96-well Nunclon microplates treated for cell cultures (Thermo Scientific, Waltham, MA, USA), and in each well 190 µL cell suspension (containing an estimative number of 2 × 10^4^ cells) was dispensed. The plates were then kept in an incubator (Revco Ultima II, from Thermo Electron Corporation, Asheville, NC, USA) at 37 °C in 5% CO_2_ atmosphere for about 24 h, to allow the cells to attach. Subsequently, the wells were treated with the nanostructured materials; 10 µL of diluted materials suspension was added to each well, for a total of 7 serial dilutions for each material, between 10 and 500 μg/mL final concentration on cells. The testing was repeated three times on three separate microplates for each compound in duplicate. Untreated wells were used as reference for 100% viability, and the cell culture media was used as blank value.

After 24 h exposure, cell growth inhibition was evaluated, as described before [66], through the colorimetric MTT test. The measurement is based on 3-(4,5-dimethylthiazol-2-yl)-2,5-diphenyltetrazolium bromide dye (MTT, from Sigma Aldrich) conversion inside the living cells, which yields its purple-coloured formazan form, detectable at 570 nm with the Synergy2 multi-plate reader (from BioTek Company, Winooski, VT, USA).

### 2.10. Modulation of Soluble Folate Receptor Alpha

An enzyme-linked immunosorbent assay (ELISA) was performed for the quantitative evaluation of the soluble folate receptor alpha, or FOLR1, from the cell culture media of the tumour cell lines subjected to treatment (FOLR1 Human Elisa kit from Mybiosource Inc., San Diego, CA, USA).

A431, DLD-1 and OVCAR3 cells were cultured for 24 h after plating on two 24-well plates, in 1 mL appropriate media for each cell line, at a concentration of 10^5^ cells/mL. After 24 h, the cell culture media was gently removed and replaced with fresh media containing a concentration of 100 µg/mL of each of the 14 tested compounds. The same concentrations were used on the second plate, to obtain duplicates.

As the tin concentration incorporated in MSU-2-AP-FA25-Sn was below the Sn quantity of MSU-2-AP-FA-Sn and much lower than in MSU-2-Sn, to evaluate the level of folate receptor, an adjustment to obtain the same tin concentration in all solutions was carried out. Therefore, the concentration of MSU-2-AP-FA-Sn was 96.7 µg/mL, and the concentration of MSU-2-Sn was 41.8 µg/mL. In addition, the Sn concentration in MSN-AP-FA25-Sn was lower than those in MSN-AP-FA-Sn and MSN-Sn, respectively. For the functional test, 75.00 µg/mL MSN-AP-FA-Sn and 41.01 µg/mL MSN-AP-FA25-Sn solutions were applied on the cells in order to obtain identical concentrations of tin.

As a reference, one well on each plate was kept untreated as a baseline colour control consisting of cell culture media without cells and without any compound. Plates were kept in the incubator for 24 h. Then, from each treated, reference or blank well the media was removed, centrifuged at 4000 rpm, aliquoted and kept at −80 °C. When all samples were harvested, they were thawed at the same time before the ELISA testing was completed.

Following the manufacturer’s instructions, the samples in duplicate (an amount of 100 µL for each well), a control consisting of cell culture media, and the standard FOLR1 protein solutions were loaded onto the coated 96-well plate and incubated at 37 °C for one hour. Then, the liquid was removed from the wells, and 100 µL of detection antibody was dispensed in each well. The plate was incubated for another hour at 37 °C, and the plate was washed three times with the wash buffer provided by the kit using an automated plate washer (DIAsource, Louvain-la-Neuve, Belgium). The plate was finally incubated for 30 min with 100 µL of detection reagent provided by the kit, and after five consecutive steps of aspiration/wash and 20 min incubation with 90 µL 3,3′,5,5′-tetramethylbenzidine (TMB) chromogenic substrate, the stop solution was added to each well and the microplate with the samples. The plate was then read immediately with the ELISA reader (Tecan Sunrise, from Tecan Group, Männedorf, Switzerland) at 450/540 nm, and the concentrations were calculated by the software (Magellan Software, Tecan Group, Männedorf, Switzerland) of the equipment.

## 3. Results and Discussion

### 3.1. Synthesis and Characterization of Starting Porous Silica Materials

**Figure 1:**
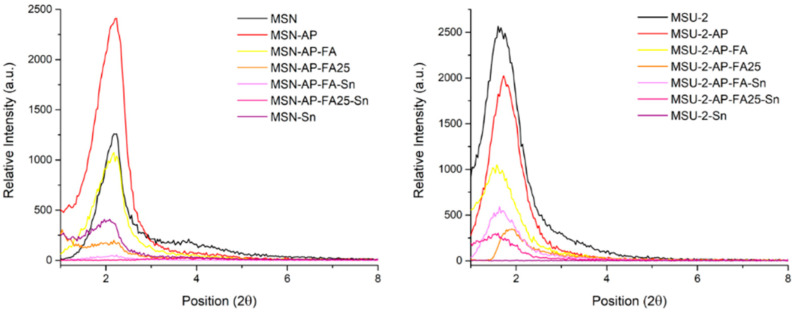
Low angle powder X-ray diffractograms of mesoporous silica nanoparticle (MSN)-based materials (**left**) and MSU-2-based materials (**right**).

Two different silica-based nanostructured materials were synthesized: mesoporous silica nanoparticles (MSNs) and a micronic spherical silica of the MSU-2 type. MSN was synthesized by the reaction of TEOS and NaOH in the presence of CTAB as surfactant with slight modification of a reported procedure [62]. The material was characterized by different methods. Powder XRD measurements showed a diffractogram with single peak of high intensity at 2θ: 2.1°, which was attributed to the 100 plane of MSN and two low-intensity peaks at 3.9° and 4.2° attributed to the 111 and 200 planes of a typical hexagonal arrangements of the pores (Figure 1).

**Figure 2:**
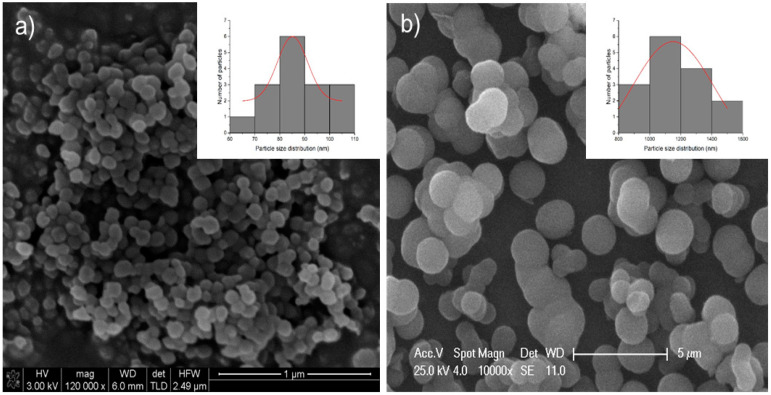
SEM images of (**a**) MSN and (**b**) MSU-2; insets represent the particle size distribution for each material calculated with ImageJ software.

**Figure 3:**
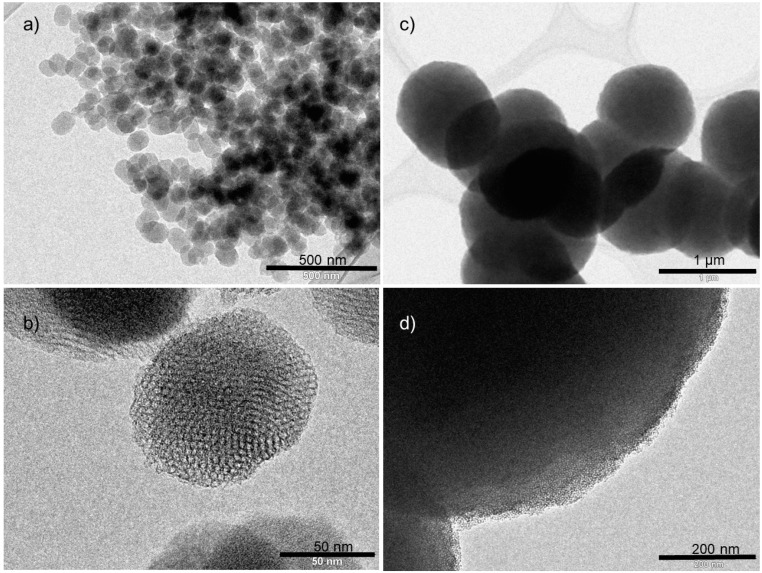
TEM images of (**a**,**b**) MSN and (**c**,**d**) MSU-2.

MSN was also analysed by SEM (Figure 2a) and TEM (Figure 3a,b). The images obtained by SEM showed a spherical morphology of the MSN particles, with a statistical study of their size by using ImageJ, which showed a particle size distribution of the MSN spheres of 87 ± 15 nm. The TEM image (Figure 3a) showed the porous nature of the particles with a hexagonal distribution of the channels of the pores as expected considering the obtained diffractogram.

MSU-2 was prepared using the method described previously [63] and which consists of the use of TEOS as silicon source and Tergitol as surfactant in the presence of an aqueous solution of NaF. The material was characterized by different methods. The diffractogram obtained by low-angle XRD for the MSU-2 sample showed a single peak of high intensity at 2θ: 1.9°, which was attributed to the 100 plane of MSU-2 (Figure 1).

It is important to note that this kind of material has a wormhole-like arrangement of the pores, which leads to a single and slightly broad peak of medium intensity as the regularity of the distance between pores is not very high. The MSU-2 silica material was also analysed by SEM to determine the size and shape of the particles. The images obtained by FEG nano-SEM (Figure 2b) showed a quasi-spherical morphology with a size distribution of 1.19 ± 0.21 μm. MSU-2 was also characterized by TEM. The micrographs clearly showed the agglomerated porous spheres (Figure 3c,d), and looking closer into the individual spheres, the porous nature of the particles and the wormhole-like arrangement of the pores can be observed (Figure 3d).

**Figure 4:**
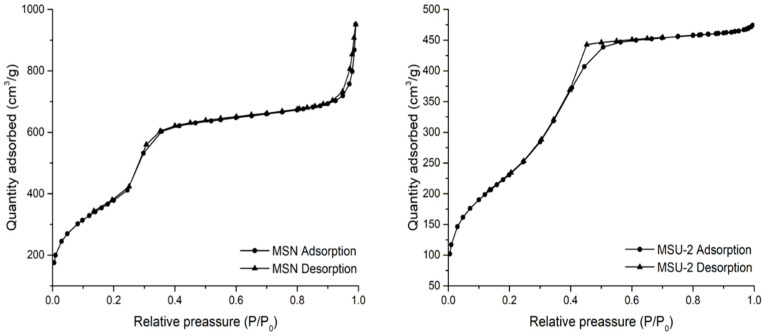
Nitrogen adsorption–desorption isotherms of MSN (**left**) and MSU-2 (**right**).

In order to determine the mesoporous nature of the host materials, a nitrogen adsorption–desorption study was carried out for both MSN and MSU-2. In the case of MSN, the obtained isotherm was between type IV and type VI (Figure 4), which is typical of mesoporous materials according to IUPAC classification [67]. In the obtained isotherm, a hysteresis loop between P/P_0_ 1.0 and about 0.8 was observed because of the capillary condensation of nitrogen into the straight pores of the system. In addition, other hysteresis loops between P/P_0_ 0.8–0.4 and 0.4–0.15 were observed, again probably, due to capillary condensation. In the case of MSU-2 nitrogen adsorption–desorption, a typical type IV isotherm with a hysteresis loop between P/P_0_ 0.8 and about 0.3 due to the capillary condensation of nitrogen was observed. The adsorption analysis allowed the determination of the BET surface areas of MSN and MSU-2, which were 1380 and 848 m^2^/g, respectively. In addition, the pore size distribution of both systems was narrow, and the pore size was determined as 3.39 and 3.41 nm for MSN and MSU-2, respectively, while the pore volumes were 1.17 and 0.72 cm^3^/g (Table 1).

### 3.2. Synthesis and Characterization of Functionalized Materials

**Scheme 1:**
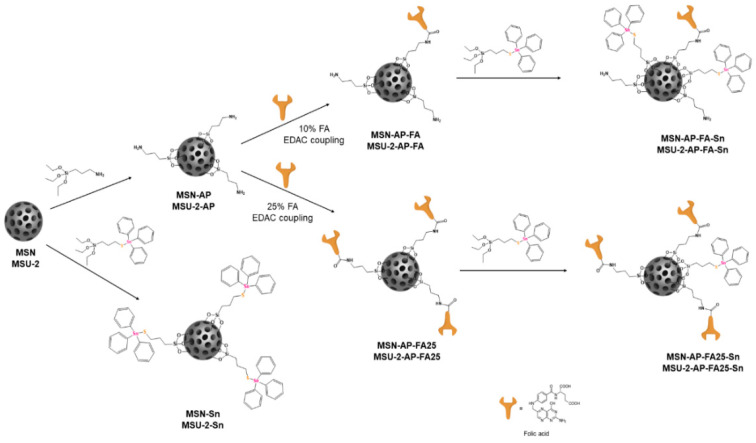
Synthesis of tin-functionalized materials incorporating folic acid of this study.

MSN and MSU-2 were functionalized with 3-aminopropyltriethoxysilane to give, respectively, MSN-AP and MSU-2-AP (Scheme 1). Subsequently, these amino-functionalized materials were also treated with 10% FA using a coupling reaction (formation of a peptide bond assisted by a carbodiimide such as EDAC) to give MSN-AP-FA or MSU-2-AP-FA (Scheme 1). Additionally, a functionalization of MSN-AP and MSU-2-AP with a higher amount (25%) of FA was carried out to give MSN-AP-FA25 or MSU-2-AP-FA25, respectively (Scheme 1). After functionalization with FA (either with 10% or 25% FA), a subsequent decoration with a Sn-based metallodrug was achieved by the protonolysis reaction of SnPh_3_{SCH_2_CH_2_CH_2_Si(OEt)_3_} and ethanol elimination to give the final materials MSN-AP-FA-Sn, MSU-2-AP-FA-Sn, MSN-AP-FA25-Sn and MSU-2-AP-FA25-Sn (Scheme 1).

**Figure 5:**
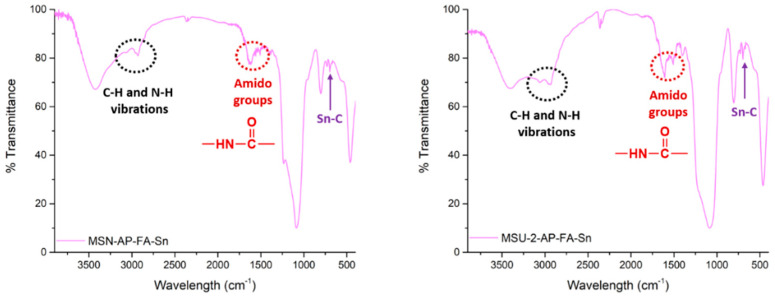
FT-IR spectra of MSN-AP-FA-Sn (**left**) and MSU-2-AP-FA-Sn (**right**).

All the synthesized materials were characterized by various techniques. Characterization by FT-IR showed incorporation of the different fragments in the structure of the silica materials. Thus, the FT-IR spectrum after functionalization with AP (MSN-AP or MSU-2-AP, see Figure S1 of Supplementary Material) showed the appearance of the signals due to C–H and *n*-H vibration between 2900 and 3200 cm^−1^. In addition, after functionalization with folic acid (MSN-AP-FA or MSU-2-AP-FA, see Figure S1 of Supplementary Material), the appearance of three new bands between 1500 and 1700 cm^−1^, corresponding to the different vibrations in the amido functional group, was observed. Finally, incorporation of the organotin(IV) fragment led to the appearance of two new signals between 600 and 700 cm^−1^ (MSN-AP-FA-Sn or MSU-2-AP-FA-Sn, Figure 5) associated with the Sn–C bonds of the SnPh_3_ moiety.

All the functionalized materials were also characterized by XRD. The low-angle diffractograms of the MSN-based functionalized materials showed, again, a major peak at 2θ of about 2.1°, as in the case of the non-functionalized MSN. However, in all the functionalized systems, this peak slightly shifted to higher angles, although the most distinguishable feature of the diffractograms is that the intensity of the major peak gradually decreased when the starting material was functionalized with APTS, FA and SnPh_3_. This is due to the partial blocking of the dispersion points of the mesostructured, which is higher when the functionalization increases with the incorporation of different fragments (Figure 1). In the case of the MSU-2-based materials, a major peak at 2θ of about 1.7° (slightly shifted to higher angles) was found for materials functionalized with AP, FA and SnPh_3_ with a clear decrease in intensity as in the case of MSN-based systems (Figure 1).

**Figure 6:**
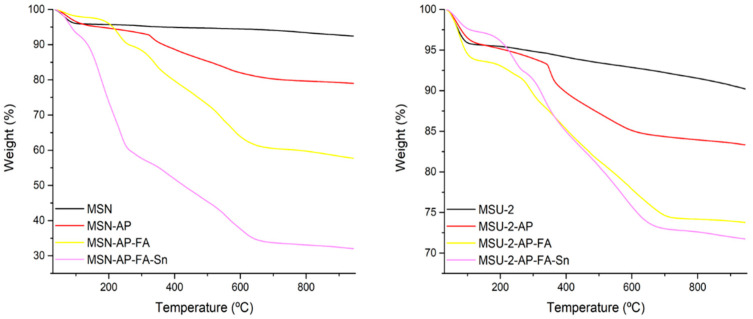
Thermogravimetric studies of MSN-based systems (**left**) and MSU-2-based materials (**right**).

The functionalized materials were also characterized by thermogravimetry (TG) to determine the quantity of the different fragments that were incorporated in the MSN or in the MSU-2 systems. The thermograms showed a clear mass loss between 120 and 650 °C, which increased upon the consecutive functionalization reactions (Table 2 and Figure 6). With the data of the thermograms, the functionalization rate was determined, observing a functionalization of about 1.31 mmol of AP per gram of MSN or 0.98 mmol of AP per gram of MSU-2. In addition, a functionalization of about 0.51 mmol of FA per gram of MSN or 0.17 mmol of FA per gram of MSU-2 was observed for MSN-AP-FA and MSU-2-AP-FA, respectively (Table 2).

In addition, the tin-functionalized materials were also characterized by both TG and XRF to determine the quantity (wt%) of supported Sn. The results showed that the quantity of Sn was between 5.7 and 14.1 (Table 2). It is important to note that the tin incorporation was very high in the free systems, MSN-Sn or MSU-2-Sn, at 13.9% and 14.1%, respectively. However, the functionalization was lower in the modified systems. Thus, those materials that have been reacted with 10% FA (MSN-AP-FA-Sn or MSU-2-AP-FA-Sn) presented higher Sn% (with values of about 7.6% and 6.1%, respectively, Table 2) compared with those which were reacted with 25% FA (MSN-AP-FA25-Sn or MSU-2-AP-FA25-Sn with functionalization of 5.7% and 5.9% Sn, respectively). Incorporation of the metal complex in the modified systems (MSN-AP-FA-Sn or MSU-2-AP-FA-Sn, MSN-AP-FA-Sn or MSU-2-AP-FA-Sn) was comparable to [26,27,33,34], if not somewhat higher [18,20,21] than, those found for some other systems based on metallodrug-functionalized silica published previously.

Although there was proof of the presence of AP, FA and/or SnPh_3_ in the structure of the functionalized MSN and MSU-2 systems, an additional NMR study of some of the key materials was carried out to further confirm incorporation of the different fragments.

**Figure 7:**
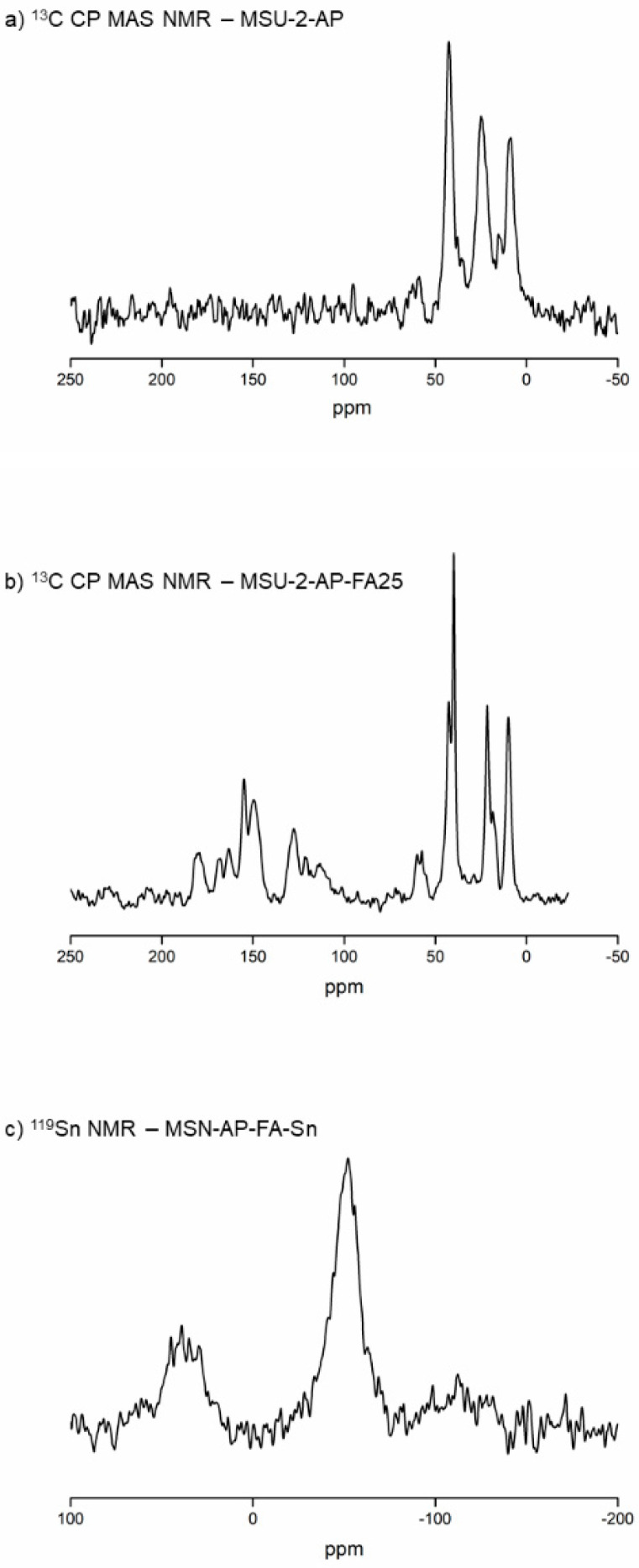
Solid-state NMR spectra of some of the studied materials: (**a**) ^13^C CP MAS NMR spectrum of MSU-2-AP, (**b**) ^13^C CP MAS NMR spectrum of MSU-2-AP-FA and (**c**) ^119^Sn NMR spectrum of MSN-AP-FA-Sn.

Thus, ^13^C CP MAS NMR spectra of MSU-2-AP and MSU-2-AP-FA25 were recorded. In the case of the ^13^C CP MAS NMR spectrum of MSU-2-AP, three broad signals between 0 and 50 ppm, assigned to the aliphatic carbon atoms of the aminopropyl chain, were observed (Figure 7a). In this context, ^13^C CP MAS NMR spectrum of MSU-2-AP-FA25 showed the same three signals between 0 and 50 ppm and a set of new signals between 120 and 180 ppm, which correspond to the aromatic C–N and C=O carbons of the FA (Figure 7b). In addition, the ^119^Sn MAS NMR spectrum of MSN-AP-FA-Sn (Figure 7c) was recorded in order to determine the incorporation of the SnPh_3_ moiety to the system. The spectrum showed an intense broad signal at −52 ppm and a lower-intensity signal at 40 ppm. The signal at −52 ppm was assigned to the tin atom bound to the S mercaptopropyl ligand, while the signal of lower intensity was due to the tin atoms of Si–O–SnPh_3_ species. The Si–O–Sn fragment was formed by slow hydrolysis of the S-Sn bond and reaction with the free silanol groups of the silica particles, as previously reported in other studies [34].

**Figure 8:**
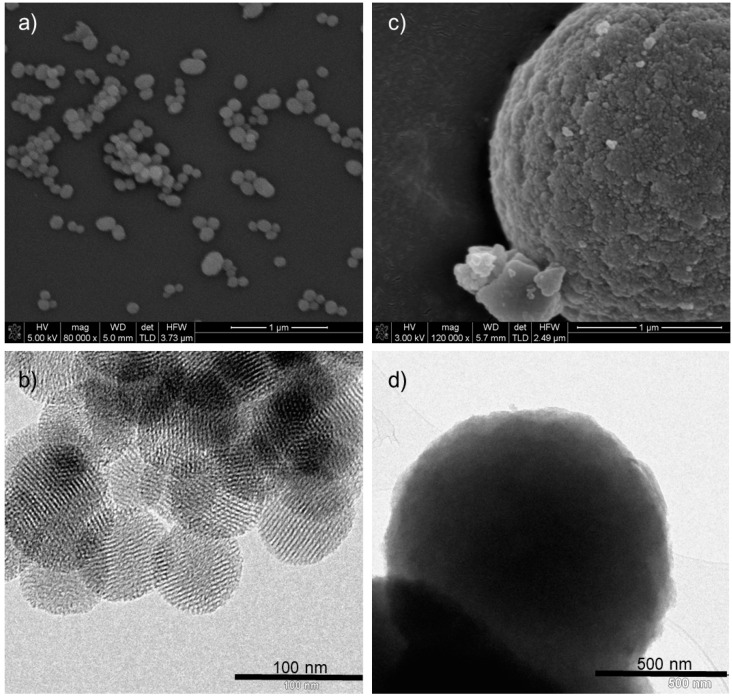
SEM and TEM images of some of the tin-functionalized materials: (**a**) SEM of MSN-AP-FA-Sn, (**b**) TEM of MSN-AP-FA-Sn, (**c**) SEM of MSU-2-AP-FA-Sn and TEM of (**d**) MSU-2-AP-FA-Sn.

The functionalized systems were also characterized by SEM and TEM observing that, after functionalization, there were no apparent changes in the morphology and particle size of the materials when compared with the unmodified MSN and MSU-2. As an example, Figure 8a shows an SEM image and Figure 8b a TEM image of MSN-AP-FA-Sn. Figure 8c, an SEM image, and Figure 8d, a TEM image of MSU-2-AP-FA-Sn, show that the morphology and particle size remained intact after functionalization steps.

Finally, the metallodrug-functionalized materials MSN-AP-FA25-Sn and MSU-2-AP-FA25-Sn were also analysed by dynamic light scattering (DLS) in order to determine their hydrodynamic size and their surface ζ-potential. The hydrodynamic size of materials MSN-AP-FA25-Sn and MSU-2-AP-FA25-Sn were of 333.1 nm (polydispersity index (PDI) of 0.29) and 2293 nm (PDI of 0.27), respectively. The sizes were in the expected range [34] and showed higher sizes than those found for TEM measurements. This is due to the functionalization of the materials with the different moieties (FA and organotin(IV) compound).

In addition, in both cases (MSN-AP-FA25-Sn and MSU-2-AP-FA25-Sn) the surface ζ-potential was negative at physiologic conditions between pH 6 and 8 as shown in Figure S10 of Supplementary Material. MSN-AP-FA25-Sn and MSU-2-AP-FA25-Sn showed isoelectric point (pI) values lower than the physiological pH (≈7.4), which give the materials a good colloidal stability that is essential for a potential use of nanomedicines in cancer therapy.

### 3.3. Anticancer Potential of the Materials Against Different Cancer Cell Lines

The two FR-α receptor-rich cancer cell lines (OVCAR-3 ovary and DLD-1 colon) and two relatively low FR-α expressing cancer cell lines (A2780 ovary and A431 squamous skin) were subjected to 24 h treatment with the nanostructured materials MSN, MSN-AP, MSN-AP-FA, MSN-AP-FA-Sn, MSN-AP-FA25, MSN-AP-FA25-Sn, MSU-2, MSU-2-AP, MSU-2-AP-FA, MSU-2-AP-FA-Sn, MSU-2-AP-FA25, MSU-2-AP-FA25-Sn, MSN-Sn and MSU-2-Sn (Figure S11 of Supplementary Material). As a reference, the same treatment was carried out on normal immortalized HaCaT keratinocytes, a cell line poor in FR-α receptors, in order to evaluate the materials selectivity (Table 3 and Figure S11 of Supplementary Material). The cell growth inhibition was completed using the MTT method (three independent measurements, in duplicate), and the compound cytotoxicity was expressed by the mathematic parameter half maximal inhibitory concentration (IC_50_), which reflects the concentration where the cell growth is reduced by half.

In the tumour cell lines, the cytotoxicity of the materials seemed to display similar behaviour, for example, between the four cell lines (OVCAR-3, DLD-1, A2780 and A431) there was a very significant correlation (two-tailed correlation, *p* < 0.0001, Spearman r value 0.973).

In all treated cell lines, the MSU-2 series was generally more cytotoxic than the MSN series. The presence of folic acid increased the cytotoxicity, especially in the materials loaded with the SnPh_3_ cytotoxic fragment. In OVCAR-3 and DLD-1 cells, the golden standard FR-α-rich lines, the cell growth inhibitory role of FA functionalization was proven by the cytotoxicity measurements. The incorporation of SnPh_3_ also increased the cytotoxicity, although less than that expected, because for other reported triphenyltin compounds or materials the cytotoxicity increased much more than in this case [12]. The incorporation of additional FA (25% vs. 10%) to MSN-AP (without the cytotoxic SnPh_3_ moiety in the structure) was not always a significant enhancer of cytotoxicity, and this indicates that the receptors from the cell outer membrane were already saturated with only 10% folic acid. This phenomenon also occurred, although attenuated, in the A431 cell line, which had fewer FA receptors on the cell surface than OVCAR-3 and DLD-1. Therefore, the highest cyotoxicity of the MSN series was displayed by MSN-AP-FA25-Sn on the FR-α-rich lines, OVCAR-3 and DLD-1 cells, apparently due to the synergy between the cytotoxic SnPh_3_ fragment and the 25% FA functionalization.

In the case of the MSU-2 series, MSU-2-AP-FA-Sn and MSU-2-AP-FA25-Sn displayed the highest cytotoxic activity with the lowest IC_50_ value, especially against OVCAR-3 and DLD-1 cells. The differences between the 10% and 25% were attenuated in the SnPh_3_-loaded structures, and the presence of folic acid significantly improved the cytotoxicity on direct comparison with the material, which only had the triphenyltin moiety (MSU-2-Sn). In A2780 and A431 folate-receptor-poor cell lines, the functionalization with FA increased the cytotoxicity but to a much lesser extent. Thus, even though the SnPh_3_-loaded, folic acid-functionalized materials MSN-AP-FA-Sn, MSN-AP-FA25-Sn, MSU-2-AP-FA-Sn and MSU-2-AP-FA25-Sn were usually the most cytotoxic, in A2780 and A431 folate-receptor-poor cell lines, they displayed similar IC_50_ values to the materials functionalized only with triphenyltin, indicating the low influence of FA in these cell lines. This did not happen in OVCAR-3 and DLD-1 in which the IC_50_ value of the most active materials MSN-AP-FA-Sn, MSN-AP-FA25-Sn, MSU-2-AP-FA-Sn and MSU-2-AP-FA25-Sn was between 2 to 8 times lower than their analogues MSN-Sn and MSU-2-Sn without FA, indicating, again, a positive influence of the incorporation of FA in the structure of the particles.

The normal HaCaT keratinocytes displayed some resistance towards the materials MSN, MSN-AP, MSN-Sn, MSU-2, MSU-2-AP and MSU-2-Sn, without folic acid; however, the FA-functionalized systems MSU-2-AP-FA and MSU-2-AP-FA25 presented selectivity towards the tumour cell lines (except A2780) versus the HaCaT cells. The best selectivity was observed in the active materials MSN-AP-FA-Sn, MSN-AP-FA25-Sn, MSU-2-AP-FA-Sn and MSU-2-AP-FA25-Sn loaded either with 10% or 25% FA and with the triphenyltin cytotoxic fragment.

### 3.4. Modulation of the Soluble Folate Receptor Alpha (FOLR1)

**Figure 9:**
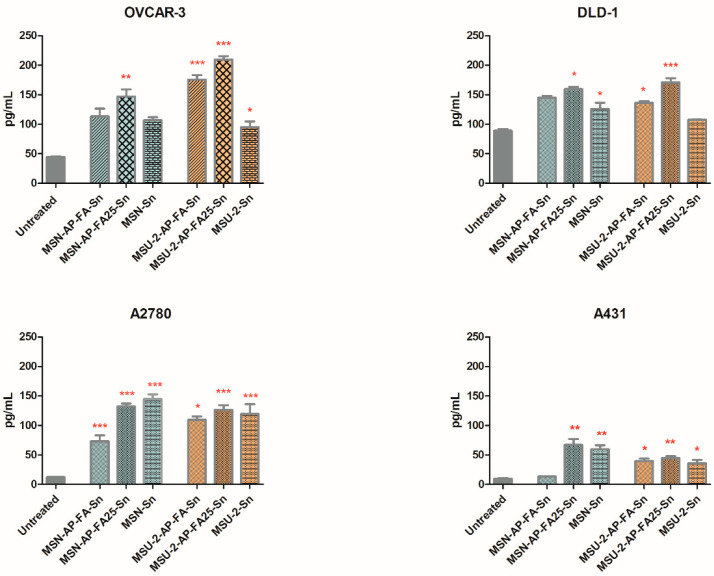
Concentration of FOLR1 secreted by tumour cells subjected to treatment with tin-containing mesoporous compounds. In the upper row the folate-receptor-positive OVCAR-3 and DLD-1 cells, in the lower row are the A2780 and A431 cells, with low folate receptor expression. Statistically significant changes in FOLR1 level vs. the untreated reference are evidenced by starring: * represents *p* < 0.05, ** *p* < 0.01 while *** *p* < 0.001. The columns with magenta borders represent the materials functionalized with 10% FA, and the blue border represents the materials functionalized with 25% FA.

Following 24 h treatment with the compounds, the concentration of the soluble folate receptor-α or FOLR1 secreted by the DLD-1, OVCAR-3, A2780 and A431 tumour cell lines was evaluated. The basal soluble FOLR1 levels, secreted by the untreated DLD-1, OVCAR-3, A2780 and A431 cells were different in each cell line (Figure 9 and Figure S12 of Supplementary Material).

Overexpression of FR-α confers an advantage for cancer cells as growth promotes folate uptake, even in poor nutrient conditions [68], and folic acid receptors play an important role in carcinogenesis, consequently FR-α is identified as an attractive therapeutic target. Early tests confirmed the presence of FR-α affects drug toxicity in vitro, but FR-α alone does not increase cancer cell sensitivity [58].

In the OVCAR-3 and DLD-1 cells that overexpress the folate receptor-α (FR-α), the basal FOLR1 level of untreated cells was superior, while in A431 and A2780 the FOLR1 production was lower. These data are in concordance with previous studies on the intracellular FOLR1 protein expression [61,69].

After 24 h exposure to the materials, at a concentration of 100 µg/mL in the cell culture media, most of the materials caused an increase, to some extent, of the FOLR1, except MSU-2, MSU-2-AP and MSN-AP, which reduced the folate receptor in DLD-1 or OVCAR-3 cells. Some of these changes were statistically significant when compared with the untreated cells. In all cell lines, MSU-2-AP-FA25-Sn and MSN-AP-FA25-Sn treatment significantly enhanced the soluble FOLR1 expression (one-way ANOVA, Bonferroni post-test, 95% confidence interval, Figure S12 of Supplementary Material).

The materials MSU-2-AP-FA-Sn and MSN-AP-FA-Sn have the capacity to significantly increase the soluble FOLR1 expression relative to the untreated cell population, even when having only 10% FA functionalization together with the triphenyltin group. One exception was in the OVCAR-3 cells, where MSU-2-AP-FA-Sn produced no significant effect (Figure 9).

Regardless of the membrane FR-α upregulation (OVCAR-3 and DLD-1 cells) or FR-α downregulation (A2780 and A431 cells), no significant difference was evidenced between the capacity of MSU-2-AP-FA25-Sn and MSU-2-AP-FA-Sn to increase the soluble FOLR1 production; therefore, the switch from 10% folic acid to 25% folic acid conferred no direct advantages in this regard. MSN-AP-25FA-Sn and MSN-AP-FA-Sn displayed the same outcome in OVCAR-3, DLD-1 and A431 cells and the material containing 25% FA, namely, MSN-AP-25FA-Sn was more active only in A2780 cells (Figure 9). This suggests that a 10% concentration of FA on the surfaces of MSU-2 or MSN-AP is a very good vector to target the tumour cell, while 25% FA is close to an oversupply.

Interestingly, MSU-2-Sn and MSN-Sn were also capable of significantly upregulating soluble FOLR1 in A2780 and A431 populations (Figure 9) with fewer folate receptors on their membrane. MSU-2-Sn also had a relevant effect on the folate-receptor-rich OVCAR-3 cells (*p* < 0.05).

In all cell lines, the presence of folic acid on the structure conferred an increase in FOLR1 secretion, and the materials with 25% FA displayed a better modulator effect than their analogues with only SnPh_3_ (without FA). This phenomenon was especially noteworthy for the pair MSU-2-AP-FA25-Sn vs. MSU-2-Sn (*p* < 0.01) in DLD-1 cells.

From the MSU-2 series, all Sn-functionalized materials, namely, MSU-2-AP-FA-Sn, MSU-2-AP-FA25-Sn and MSU-2-Sn, extensively increased the FOLR1 secreted by A431 and A2780 cells (*p* < 0.01), while the FOLR1 level was comparable for all Sn-functionalized compounds (*p* > 0.05, no statistical significance). In these two cell lines with reduced FR-α expression (A431 and A2780), the presence of 10% FA or 25% FA on the material surface did not increase their activity compared with MSU-2-Sn, which contains no folic acid, and is a rational consequence of the lack of connection between FA and its receptor FR-α. The materials without both the FA and the cytotoxic organotin fragment did not significantly modify the FOLR1 level, with the exception of MSU-2-AP in the A431 cell line.

When the mesoporous materials MSU-2 and MSN were functionalized with only folic acid, no remarkable changes in FOLR1 production (Figure S12 of Supplementary Material) were generated either by the lower concentration (10% FA), or by the higher concentration (25% FA), on direct comparison with the untreated cells, with the unique exception of MSU-2-AP-FA in the FR-α poor A2780 cells. As FOLR1 modulators, the FA-functionalized materials were usually similar to their precursors. Therefore, from the viewpoint of the biological function, the presence of FA on the nanostructure surface led to better results only together with the incorporation of the tin-containing cytotoxic fragment.

The level of secreted FOLR1 (Figure 9 and Figure S12 of Supplementary Material) correlated well with the IC_50_ values (Table 3) in both the MSN and MSU-2 series for the folate-rich cell lines OVCAR-3 (nonparametric two-tailed Spearman correlation, *p* value 0.0186) and DLD-1 (*p* value 0.0213). This correlation also occurred in A2780 (*p* value 0.0039) and A431 (*p* value 0.0055) cells with under-expressed folate receptor on their membrane. Therefore, the MSN and MSU-2 material cytotoxicity against tumour cells relies on their capacity to incise the intracellular or membrane-anchored folate alpha-receptors, influencing the soluble FOLR1 shedding. The increment of the soluble FOLR1 secretion by the tumour cells is, presumably, possible by cutting the glycosyl-phosphatidylinositol anchor of membrane FR-α and by the release of intracellular FR-α; therefore, it was essential to functionalize the nanostructured materials with the cytotoxic triphenyltin group, and with a relatively high concentrations of folic acid (over 25%). Therefore, in the structure of the MSN- and MSU-2-based materials, functionalization with folic acid has a multifunctional role beyond targeting the folate receptor-rich cells, firstly to influence the soluble FOLR1 secretion of the tumour cells, and then to inhibit their proliferation. Finally, while in the case of the cytotoxic action of the systems, the size and shape of the silica-based materials seemed to influence the cytotoxic action. In the case of FOLR1 modulation, the much lower size of MSN materials compared with MSU-2 systems did not seem to have a significant impact on the potential targeting. In general, all the analogue systems behaved similarly with no notable changes in the FOLR1 modulation, even though the porous systems were very different.

## 4. Conclusions

Incorporation of folic acid in the structure of the two different mesoporous silica-based materials, namely MSU-2 and MSN, has been confirmed and, together with the functionalization with an organotin(IV) derivative, is an interesting synthetic alternative to design materials able to target cancer cells that overexpress folate receptors, such as OVCAR-3 and DLD-1. In general, it has been observed that the tin-functionalized MSU-2 systems are more cytotoxic than their analogues based on MSN. In addition, functionalization with higher amounts of folic acid leads to higher cytotoxicity and therapeutic potential, especially in cells overexpressing folate receptors. Furthermore, the study of the modulation of the FOLR1 receptor promoted by the different studied silica-based systems showed that the level of secreted FOLR1 correlated well with the IC_50_ values in both MSN and MSU-2 series. In addition, functionalization with folic acid had a multifunctional role beyond targeting the folate receptor-rich cells, the materials with folic acid and the organotin(IV) fragment in their structure influenced the soluble FOLR1 secretion of the tumour cells and then inhibited their proliferation.

In summary, this strategy based on the incorporation of folic acid and a SnPh_3_ moiety results in potent systems against different cancer cell lines and should be an interesting field of study for other metallodrug-functionalized silica-based systems. Thus, fine tuning of the nanostructured silica-based systems with the appropriate FA quantities and metallodrug structure may lead to more effective systems for the therapeutic treatment of different tumours composed of cells overexpressing folate receptors.

## Figures and Tables

**Table 1:** Textural parameters of MSN and MSU-2 obtained by nitrogen adsorption–desorption analysis.

Material	S_BET_ (m^2^/g)	Pore Volume (cm^3^/g)	Pore Diameter (nm)
MSN	1380	1.17	3.39
MSU-2	848	0.72	3.41

**Table 2:** Functionalization rates for some of the studied materials determined by TG or XRF.

Material	AP (TG) mmol/g	FA (TG) mmol/g	%Sn ± SD (XRF)
MSN-AP-FA-Sn	1.31	0.51	7.56 ± 0.03
MSU-2-AP-FA-Sn	0.98	0.17	6.12 ± 0.03
MSN-AP-FA25-Sn	n.a.^a^	n.a.	5.68 ± 0.03
MSU-2-AP-FA25-Sn	n.a.	n.a.	5.91 ± 0.04
MSN-Sn	-	-	13.90 ± 0.03
MSU-2-Sn	-	-	14.10 ± 0.03

^a^ Not analysed.

**Table 3:** The half maximal inhibitory concentrations (IC_50_) of the MSN and MSU-2 materials; IC_50_ were derived from the nonlinear dose–response curves, in the 95% confidence interval; values expressed as mean ± standard deviation, in μg/mL.

Material/Cell Line	OVCAR-3	DLD-1	A2780	A431	HaCaT
MSN	267.11 ± 15.14	>500	>500	>500	440.45 ±17.73
MSN-AP	223.09 ± 18.55	>500	>500	>500	358.40 ± 41.88
MSN-AP-FA	177.78 ± 11.79	392.07 ± 24.10	>500	429.55 ± 34.03	360.81 ± 32.62
MSN-AP-FA25	182.65 ± 20.32	327.53 ± 17.05	341.91 ± 23.31	432.76 ± 28.02	342.65 ± 20.05
MSN-AP-FA-Sn	50.36 ± 3.82	82.32 ± 9.44	155.19 ± 6.59	102.5 ± 7.71	134.18 ± 15.91
MSN-AP-FA25-Sn	22.49 ± 1.43	67.70 ± 4.08	143.63 ± 9.77	94.01 ± 5.62	152.80 ± 9.88
MSN-Sn	101.55 ± 2.11	184.03 ± 25.64	198.38 ± 9.99	87.81 ± 13.47	123.98 ± 20.41
MSU-2	183.71 ± 26.85	266.43 ± 10.27	348.46 ± 29.72	112.40 ± 20.13	301.92 ± 16.12
MSU-2-AP	164.80 ± 15.00	235.55 ± 9.04	329.25 ± 37.33	74.26 ± 7.03	256.72 ± 30.52
MSU-2-AP-FA	96.75 ± 8.31	189.25 ± 26.85	296.54 ± 20.54	90.18 ± 13.86	233.20 ± 16.90
MSU-2-AP-FA25	91.14 ± 10.64	103.23 ± 8.00	288.62 ± 12.27	88.33 ± 4.6	239.48 ± 17.82
MSU-2-AP-FA-Sn	21.02 ± 4.16	49.60 ± 8.3	146.05 ± 9.08	53.78 ± 11.25	144.48 ± 6.81
MSU-2-AP-FA25-Sn	9.35 ± 1.13	20.64 ± 0.88	96.83 ± 6.42	65.30 ± 5.80	115.10 ± 9.72
MSU-2-Sn	79.61 ± 7.55	107.04 ± 11.45	181.98 ± 10.69	59.19 ± 18.97	124.32 ± 10.18

## Supplementary Materials

The following are available online at https://www.mdpi.com/1999-4923/12/6/512/s1, Figure S1. FT-IR spectra of MSN, MSN-AP, MSN-AP-FA, MSN-AP-FA-Sn, MSU-2, MSU-2-AP, MSU-2-AP-FA and MSU-2-AP-FA-Sn. Figures S2–S9. TG and DSC analyses of MSN, MSN-AP, MSN-AP-FA, MSN-AP-FA-Sn, MSU-2, MSU-2-AP, MSU-2-AP-FA and MSU-2-AP-FA-Sn, respectively. Figure S10. Z-potential measurements between pH 3 and pH 12 for MSN-AP-FA25-Sn and MSU-2-AP-FA25-Sn. Figure S11. Nonlinear dose–response relationship between the concentration vs. the cell growth inhibition following 24 h treatment with MSU-2 and MSN materials in vitro. Figure S12. Concentration of FOLR1 secreted by tumour cells subjected to treatment with mesoporous compounds. In the upper row the folate receptor-positive DLD-1 and OVCAR-3 cells, in the lower row are the A2780 and A431 cells, with low folate receptor expression.

## References

[1] Worldwide Cancer Statistics. https://www.cancerresearchuk.org/health-professional/cancer-statistics/worldwide-cancer.

[2] Cancer Tomorrow. http://gco.iarc.fr/tomorrow/home.

[3] Jackson S.E., Chester J.D. (2015). Personalised cancer medicine. Int. J. Cancer.

[4] Dumbrava E.I., Meric-Bernstam F. (2018). Personalized cancer therapy—Leveraging a knowledge base for clinical decision-making. Cold Spring Harb. Mol. Case Stud..

[5] Chen H.H.W., Kuo M.T. (2017). Improving radiotherapy in cancer treatment: Promises and challenges. Oncotarget.

[6] Metcalfe C., Friedman L.S., Hager J.H. (2018). Hormone-Targeted Therapy and Resistance. Annu. Rev. Cancer Biol..

[7] Schirrmacher V. (2019). From chemotherapy to biological therapy: A review of novel concepts to reduce the side effects of systemic cancer treatment. Int. J. Oncol..

[8] Rosenberg B., VanCamp L. (1970). The successful regression of large solid sarcoma 180 tumors by platinum compounds. Cancer Res..

[9] Ndagi U., Mhlongo N., Soliman M.E. (2017). Metal complexes in cancer therapy—An update from drug design perspective. Drug. Des. Dev. Ther..

[10] Mjos K.D., Orvig C. (2014). Metallodrugs in medicinal inorganic chemistry. Chem. Rev..

[11] Wani W.A., Prashar S., Shreaz S., Gómez-Ruiz S. (2016). Nanostructured materials functionalized with metal complexes: In search of alternatives for administering anticancer metallodrugs. Coord. Chem. Rev..

[12] Ellahioui Y., Prashar S., Gómez-Ruiz S. (2017). Anticancer Applications and Recent Investigations of Metallodrugs Based on Gallium, Tin and Titanium. Inorganics.

[13] Poursharifi M., Wlodarczyk M.T., Mieszawska A.J. (2019). Nano-Based Systems and Biomacromolecules as Carriers for Metallodrugs in Anticancer Therapy. Inorganics.

[14] Ellahioui Y., Prashar S., Gómez-Ruiz S. (2016). A Short Overview on the Biomedical Applications of Silica, Alumina and Calcium Phosphate-based Nanostructured Materials. Curr. Med. Chem..

[15] Pérez-Quintanilla D., Gómez-Ruiz S., Žižak Ž., Sierra I., Prashar S., del Hierro I., Fajardo M., Juranić Z.D., Kaluđerović G.N. (2009). A New Generation of Anticancer Drugs: Mesoporous Materials Modified with Titanocene Complexes. Chem. Eur. J..

[16] Kaluderović G.N., Pérez-Quintanilla D., Zizak Z., Juranić Z.D., Gómez-Ruiz S. (2010). Improvement of cytotoxicity of titanocene-functionalized mesoporous materials by the increase of the titanium content. Dalton Trans..

[17] Kaluđerović G.N., Pérez-Quintanilla D., Sierra I., Prashar S., del Hierro I., Žižak Ž., Juranić Z.D., Fajardo M., Gómez-Ruiz S. (2010). Study of the influence of the metal complex on the cytotoxic activity of titanocene-functionalized mesoporous materials. J. Mater. Chem..

[18] García-Peñas A., Gómez-Ruiz S., Pérez-Quintanilla D., Paschke R., Sierra I., Prashar S., del Hierro I., Kaluđerović G.N. (2012). Study of the cytotoxicity and particle action in human cancer cells of titanocene-functionalized materials with potential application against tumors. J. Inorg. Biochem..

[19] Bulatović M.Z., Maksimović-Ivanić D., Bensing C., Gómez-Ruiz S., Steinborn D., Schmidt H., Mojić M., Korać A., Golić I., Pérez-Quintanilla D. (2014). Organotin(IV)-loaded mesoporous silica as a biocompatible strategy in cancer treatment. Angew. Chem. Int. Ed. Engl..

[20] Ceballos-Torres J., Virag P., Cenariu M., Prashar S., Fajardo M., Fischer-Fodor E., Gómez-Ruiz S. (2014). Anti-cancer Applications of Titanocene-Functionalised Nanostructured Systems: An Insight into Cell Death Mechanisms. Chem. Eur. J..

[21] Ceballos-Torres J., Prashar S., Fajardo M., Chicca A., Gertsch J., Pinar A.B., Gómez-Ruiz S. (2015). Ether-Substituted Group 4 Metallocene Complexes: Cytostatic Effects and Applications in Ethylene Polymerization. Organometallics.

[22] Bensing C., Mojić M., Gómez-Ruiz S., Carralero S., Dojčinović B., Maksimović-Ivanić D., Mijatović S., Kaluđerović G.N. (2016). Evaluation of functionalized mesoporous silica SBA-15 as a carrier system for Ph_3_Sn(CH_2_)_3_OH against the A2780 ovarian carcinoma cell line. Dalton Trans..

[23] Díaz-García D., Cenariu D., Pérez Y., Cruz P., del Hierro I., Prashar S., Fischer-Fodor E., Gómez-Ruiz S. (2018). Modulation of the mechanism of apoptosis in cancer cell lines by treatment with silica-based nanostructured materials functionalized with different metallodrugs. Dalton Trans..

[24] Gómez-Ruiz S., García-Peñas A., Prashar S., Rodríguez-Diéguez A., Fischer-Fodor E. (2018). Anticancer Applications of Nanostructured Silica-Based Materials Functionalized with Titanocene Derivatives: Induction of Cell Death Mechanism through TNFR1 Modulation. Materials.

[25] Del Hierro I., Gómez-Ruiz S., Pérez Y., Cruz P., Prashar S., Fajardo M. (2018). Mesoporous SBA-15 modified with titanocene complexes and ionic liquids: Interactions with DNA and other molecules of biological interest studied by solid state electrochemical techniques. Dalton Trans..

[26] Díaz-García D., Ardiles P.R., Prashar S., Rodríguez-Diéguez A., Páez P.L., Gómez-Ruiz S. (2019). Preparation and Study of the Antibacterial Applications and Oxidative Stress Induction of Copper Maleamate-Functionalized Mesoporous Silica Nanoparticles. Pharmaceutics.

[27] Ellahioui Y., Patra M., Mari C., Kaabi R., Karges J., Gasser G., Gómez-Ruiz S. (2019). Mesoporous silica nanoparticles functionalised with a photoactive ruthenium(ii) complex: Exploring the formulation of a metal-based photodynamic therapy photosensitiser. Dalton Trans..

[28] Edeler D., Kaluđerović M.R., Dojčinović B., Schmidt H., Kaluđerović G.N. (2016). SBA-15 mesoporous silica particles loaded with cisplatin induce senescence in B16F10 cells. RSC Adv..

[29] Rojas S., Carmona F.J., Barea E., Maldonado C.R. (2017). Inorganic mesoporous silicas as vehicles of two novel anthracene-based ruthenium metalloarenes. J. Inorg. Biochem..

[30] Edeler D., Arlt S., Petković V., Ludwig G., Drača D., Maksimović-Ivanić D., Mijatović S., Kaluđerović G.N. (2018). Delivery of [Ru(η^6^-p-cymene)Cl_2_{Ph_2_P(CH_2_)_3_SPh-κP}] using unfunctionalized and mercapto functionalized SBA-15 mesoporous silica: Preparation, characterization and in vitro study. J. Inorg. Biochem..

[31] Maksimović-Ivanić D., Bulatović M., Edeler D., Bensing C., Golić I., Korać A., Kaluđerović G.N., Mijatović S. (2019). The interaction between SBA-15 derivative loaded with Ph_3_Sn(CH_2_)_6_OH and human melanoma A375 cell line: Uptake and stem phenotype loss. J. Biol. Inorg. Chem..

[32] Edeler D., Drača D., Petković V., Natalio F., Maksimović-Ivanić D., Mijatović S., Schmidt H., Kaluđerović G.N. (2019). Impact of the mesoporous silica SBA-15 functionalization on the mode of action of Ph_3_Sn(CH_2_)_6_OH. Mater. Sci. Eng. C.

[33] Díaz-García D., Sommerova L., Martisova A., Skoupilova H., Prashar S., Vaculovic T., Kanicky V., del Hierro I., Hrstka R., Gómez-Ruiz S. (2020). Mesoporous silica nanoparticles functionalized with a dialkoxide diorganotin(IV) compound: In search of more selective systems against cancer cells. Microporous Mesoporous Mater..

[34] Ovejero Paredes K., Díaz-García D., García-Almodóvar V., Lozano Chamizo L., Marciello M., Díaz-Sánchez M., Prashar S., Gómez-Ruiz S., Filice M. (2020). Multifunctional Silica-Based Nanoparticles with Controlled Release of Organotin Metallodrug for Targeted Theranosis of Breast Cancer. Cancers.

[35] Fan J., Fang G., Wang X., Zeng F., Xiang Y., Wu S. (2011). Targeted anticancer prodrug with mesoporous silica nanoparticles as vehicles. Nanotechnology.

[36] Lu J., Li Z., Zink J.I., Tamanoi F. (2012). In vivo tumor suppression efficacy of mesoporous silica nanoparticles-based drug-delivery system: Enhanced efficacy by folate modification. Nanomedicine.

[37] Khosravian P., Shafiee Ardestani M., Khoobi M., Ostad S.N., Dorkoosh F.A., Akbari Javar H., Amanlou M. (2016). Mesoporous silica nanoparticles functionalized with folic acid/methionine for active targeted delivery of docetaxel. Onco Targets Ther..

[38] Knežević N.Ž., Mrđanović J., Borišev I., Milenković S., Janaćković Đ., Cunin F., Djordjevic A. (2016). Hydroxylated fullerene-capped, vinblastine-loaded folic acid-functionalized mesoporous silica nanoparticles for targeted anticancer therapy. RSC Adv..

[39] Wang J., Wang Y., Liu Q., Yang L., Zhu R., Yu C., Wang S. (2016). Rational Design of Multifunctional Dendritic Mesoporous Silica Nanoparticles to Load Curcumin and Enhance Efficacy for Breast Cancer Therapy. ACS Appl. Mater. Interfaces.

[40] Khattabi A.M., Talib W.H., Alqdeimat D.A. (2017). A targeted drug delivery system of anti-cancer agents based on folic acid-cyclodextrin-long polymer functionalized silica nanoparticles. J. Drug Deliv. Sci. Technol..

[41] Liu Q., Xu N., Liu L., Li J., Zhang Y., Shen C., Shezad K., Zhang L., Zhu J., Tao J. (2017). Dacarbazine-Loaded Hollow Mesoporous Silica Nanoparticles Grafted with Folic Acid for Enhancing Antimetastatic Melanoma Response. ACS Appl. Mater. Interfaces.

[42] Qu W., Meng B., Yu Y., Wang S. (2018). Folic acid-conjugated mesoporous silica nanoparticles for enhanced therapeutic efficacy of topotecan in retina cancers. Int. J. Nanomed..

[43] Shen S., Li Y., Xiao Y., Zhao Z., Zhang C., Wang J., Li H., Liu F., He N., Yuan Y. (2018). Folate-conjugated nanobubbles selectively target and kill cancer cells via ultrasound-triggered intracellular explosion. Biomaterials.

[44] Liu Y., Zong Y., Yang Z., Luo M., Li G., Yingsa W., Cao Y., Xiao M., Kong T., He J. (2019). Dual-Targeted Controlled Delivery Based on Folic Acid Modified Pectin-Based Nanoparticles for Combination Therapy of Liver Cancer. ACS Sustain. Chem. Eng..

[45] Poltavets Y.I., Zhirnik A.S., Zavarzina V.V., Semochkina Y.P., Shuvatova V.G., Krasheninnikova A.A., Aleshin S.V., Dronov D.O., Vorontsov E.A., Balabanyan V.Y. (2019). In vitro anticancer activity of folate-modified docetaxel-loaded PLGA nanoparticles against drug-sensitive and multidrug-resistant cancer cells. Cancer Nanotechnol..

[46] De Oliveira Silva J., Fernandes R.S., Ramos Oda C.M., Ferreira T.H., Machado Botelho A.F., Martins Melo M., de Miranda M.C., Assis Gomes D., Dantas Cassali G., Townsend D.M. (2019). Folate-coated, long-circulating and pH-sensitive liposomes enhance doxorubicin antitumor effect in a breast cancer animal model. Biomed. Pharmacother..

[47] Iturrioz-Rodríguez N., Correa-Duarte M.A., Fanarraga M.L. (2019). Controlled drug delivery systems for cancer based on mesoporous silica nanoparticles. Int. J. Nanomed..

[48] Malekmohammadi S., Hadadzadeh H., Amirghofran Z. (2018). Preparation of folic acid-conjugated dendritic mesoporous silica nanoparticles for pH-controlled release and targeted delivery of a cyclometallated gold(III) complex as an antitumor agent. J. Mol. Liq..

[49] Fernández M., Javaid F., Chudasama V. (2018). Advances in targeting the folate receptor in the treatment/imaging of cancers. Chem. Sci..

[50] Hartmann L.C., Keeney G.L., Lingle W.L., Christianson T.J.H., Varghese B., Hillman D., Oberg A.L., Low P.S. (2007). Folate receptor overexpression is associated with poor outcome in breast cancer. Int. J. Cancer.

[51] Wibowo A.S., Singh M., Reeder K.M., Carter J.J., Kovach A.R., Meng W., Ratnam M., Zhang F., Dann C.E. (2013). Structures of human folate receptors reveal biological trafficking states and diversity in folate and antifolate recognition. Proc. Natl. Acad. Sci. USA.

[52] Yi Y.-S. (2016). Folate Receptor-Targeted Diagnostics and Therapeutics for Inflammatory Diseases. Immune Netw..

[53] Jelovac D., Armstrong D.K. (2011). Recent progress in the diagnosis and treatment of ovarian cancer. CA Cancer J. Clin..

[54] Hansen M.F., Greibe E., Skovbjerg S., Rohde S., Kristensen A.C.M., Jensen T.R., Stentoft C., Kjær K.H., Kronborg C.S., Martensen P.M. (2015). Folic acid mediates activation of the pro-oncogene STAT3 via the Folate Receptor alpha. Cell Signal..

[55] Kelemen L.E., Brenton J.D., Parkinson C., Whitaker H.C., Piskorz A.M., Csizmadi I., Robson P.J. (2014). Conditions Associated with Circulating Tumor-Associated Folate Receptor 1 Protein in Healthy Men and Women. PLoS ONE.

[56] Basal E., Eghbali-Fatourechi G.Z., Kalli K.R., Hartmann L.C., Goodman K.M., Goode E.L., Kamen B.A., Low P.S., Knutson K.L. (2009). Functional folate receptor alpha is elevated in the blood of ovarian cancer patients. PLoS ONE.

[57] Leung F., Dimitromanolakis A., Kobayashi H., Diamandis E.P., Kulasingam V. (2013). Folate-receptor 1 (FOLR1) protein is elevated in the serum of ovarian cancer patients. Clin. Biochem..

[58] Sen S., Erba E., D’Incalci M., Bottero F., Canevari S., Tomassetti A. (1996). Role of membrane folate-binding protein in the cytotoxicity of 5,10-dideazatetrahydrofolic acid in human ovarian carcinoma cell lines in vitro. Br. J. Cancer.

[59] Chen L., Peng M., Li N., Song Q., Yao Y., Xu B., Liu H., Ruan P. (2018). Combined use of EpCAM and FRα enables the high-efficiency capture of circulating tumor cells in non-small cell lung cancer. Sci. Rep..

[60] Tsukihara H., Tsunekuni K., Takechi T. (2016). Folic Acid-Metabolizing Enzymes Regulate the Antitumor Effect of 5-Fluoro-2′-Deoxyuridine in Colorectal Cancer Cell Lines. PLoS ONE.

[61] Theti D.S., Jackman A.L. (2004). The role of alpha-folate receptor-mediated transport in the antitumor activity of antifolate drugs. Clin. Cancer Res..

[62] Zhao Y., Trewyn B.G., Slowing I.I., Lin V.S.-Y. (2009). Mesoporous Silica Nanoparticle-Based Double Drug Delivery System for Glucose-Responsive Controlled Release of Insulin and Cyclic AMP. J. Am. Chem. Soc..

[63] Pérez-Quintanilla D., Sánchez A., del Hierro I., Fajardo M., Sierra I. (2009). Synthesis and characterization of novel mesoporous silicas of the MSU-X family for environmental applications. J. Nanosci. Nanotechnol..

[64] Bollu V.S., Barui A.K., Mondal S.K., Prashar S., Fajardo M., Briones D., Rodríguez-Diéguez A., Patra C.R., Gómez-Ruiz S. (2016). Curcumin-loaded silica-based mesoporous materials: Synthesis, characterization and cytotoxic properties against cancer cells. Mater. Sci. Eng. C Mater. Biol. Appl..

[65] Kotcherlakota R., Barui A.K., Prashar S., Fajardo M., Briones D., Rodríguez-Diéguez A., Patra C.R., Gómez-Ruiz S. (2016). Curcumin loaded mesoporous silica: An effective drug delivery system for cancer treatment. Biomater. Sci..

[66] Fernández B., Oyarzabal I., Fischer-Fodor E., Macavei S., Sánchez I., Seco J.M., Gómez-Ruiz S., Rodríguez-Diéguez A. (2016). Multifunctional applications of a dysprosium-based metal–organic chain with single-ion magnet behaviour. CrystEngComm.

[67] Thommes M., Kaneko K., Neimark A.V., Olivier J.P., Rodriguez-Reinoso F., Rouquerol J., Sing K.S.W. (2015). Physisorption of gases, with special reference to the evaluation of surface area and pore size distribution (IUPAC Technical Report). Pure Appl. Chem..

[68] Dhanasekaran S. (2019). Augmented cytotoxic effects of paclitaxel by curcumin induced overexpression of folate receptor-α for enhanced targeted drug delivery in HeLa cells. Phytomedicine.

[69] Patel N.R., Piroyan A., Ganta S., Morse A.B., Candiloro K.M., Solon A.L., Nack A.H., Galati C.A., Bora C., Maglaty M.A. (2018). In Vitro and In Vivo evaluation of a novel folate-targeted theranostic nanoemulsion of docetaxel for imaging and improved anticancer activity against ovarian cancers. Cancer Biol. Ther..

