# Supplementary material for: Role of Folic Acid in the Therapeutic Action of Nanostructured Porous Silica Functionalized with Organotin(IV) Compounds against Different Cancer Cell Lines
Source: Pharmaceutics. 2020 Jun 3;12(6):512. doi: 10.3390/pharmaceutics12060512 (PMC7355810; doi:10.3390/pharmaceutics12060512)
Supplement: Supplementary file 1 [file pharmaceutics-12-00512-s001.pdf]

pharmaceutics

Supplementary Materials: Role of Folic Acid in the Therapeutic Action of Nanostructured Porous Silica Functionalized with Organotin(IV) Compounds Against Different Cancer Cell Lines

Diana Díaz-García, Karla Montalbán-Hernández, Irene Mena-Palomo, Patriciu Achimas-Cadariu, Antonio Rodríguez-Diéguez, Eduardo López-Collazo, Sanjiv Prashar, Karina Ovejero Paredes, Marco Filice, Eva Fischer-Fodor and Santiago Gómez-Ruiz

TABLE OF CONTENTS OF SUPPLEMENTARY MATERIAL

This supplementary material contains:

1) FT-IR spectra of MSN. MSU-2, MSN-AP, MSU-2-AP, MSN-AP-FA and MSU-2-AP-FA (Figure S1A-F)
2) TG and DSC analysis of MSN. MSU-2, MSN-AP, MSU-2-AP, MSN-AP-FA, MSU-2-AP-FA, MSN-AP-FA-Sn and MSU-2-AP-FA-Sn. (Figures S2-S9 respectively)
3) Z-potential measurmenets (Figure S10)
4) Curves dose-response (Figure S11)
5) Concentration of FOLR1 secreted by tumour cells subjected to treatment (Figure S12)

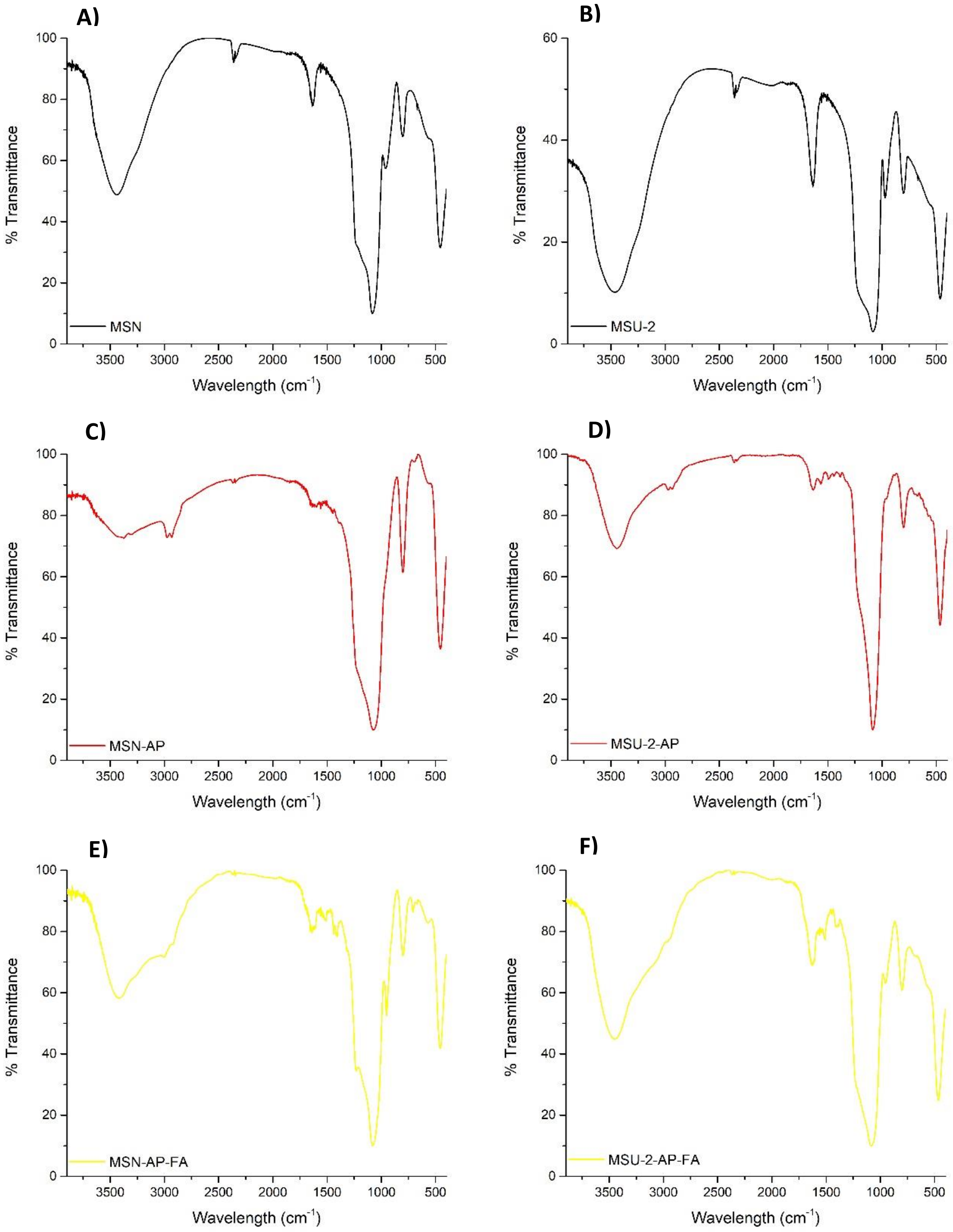

Figure S1. FT-IR spectra of the starting silica materials (A and B), functionalized with AP (C and D) and after the FA incorporation (E and F).

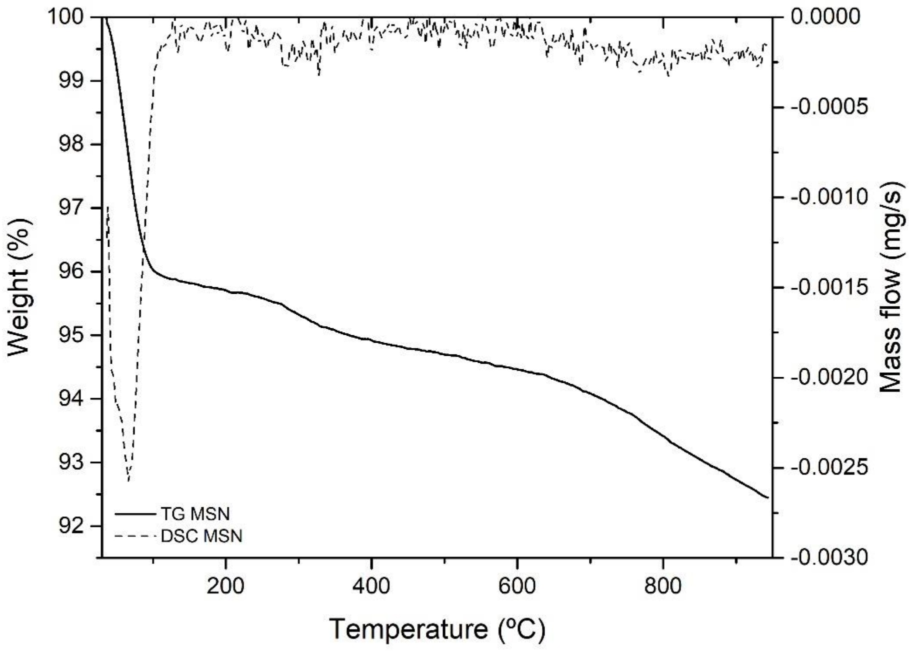

Figure S2. TG-DSC of the starting material MSN.

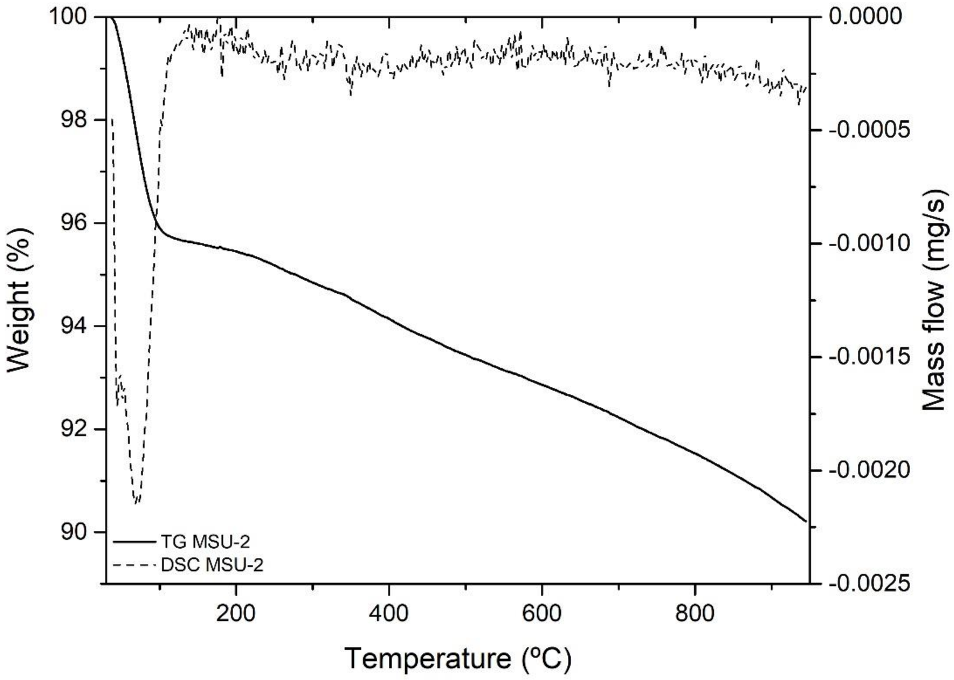

Figure S3. TG-DSC of the starting material MSU-2.

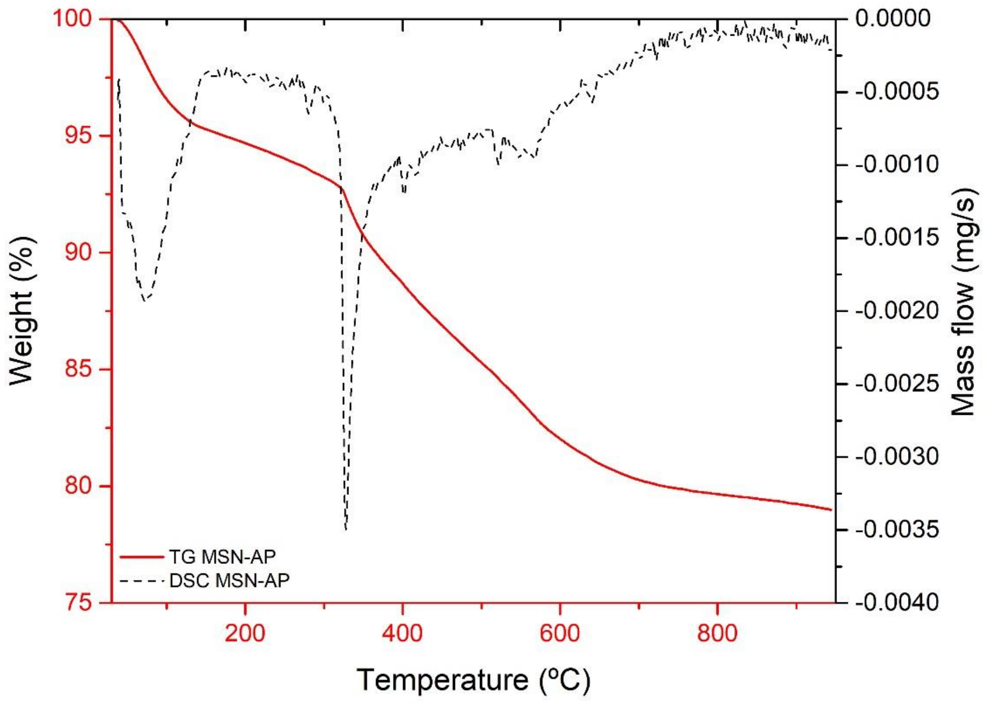

Figure S4. TG-DSC of MSN-AP.

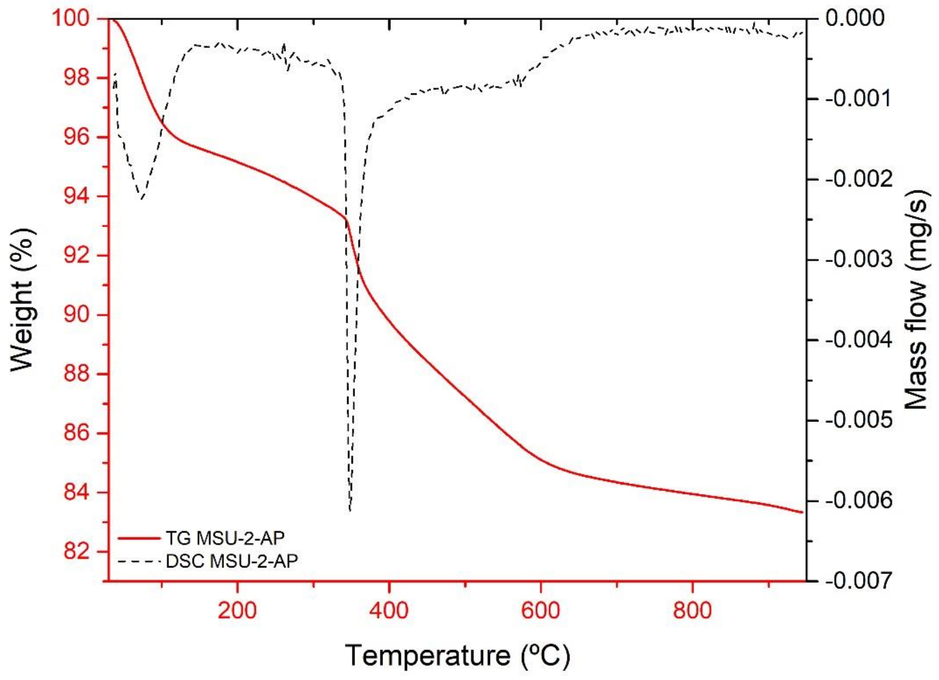

Figure S5. TG-DSC of MSU-2-AP.

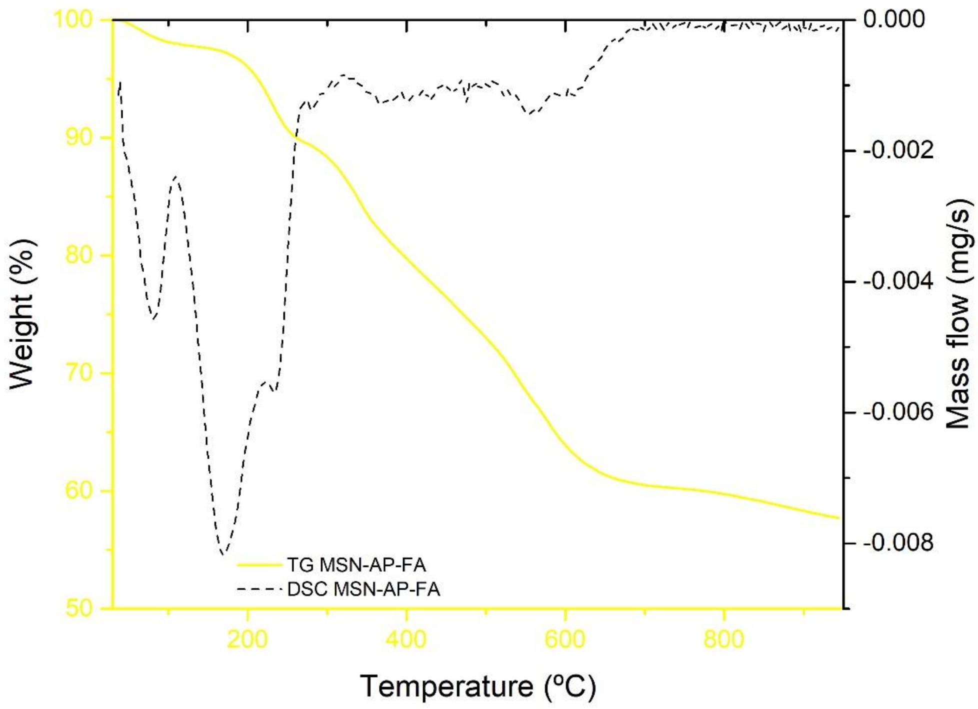

Figure S6. TG-DSC of MSN-AP-FA.

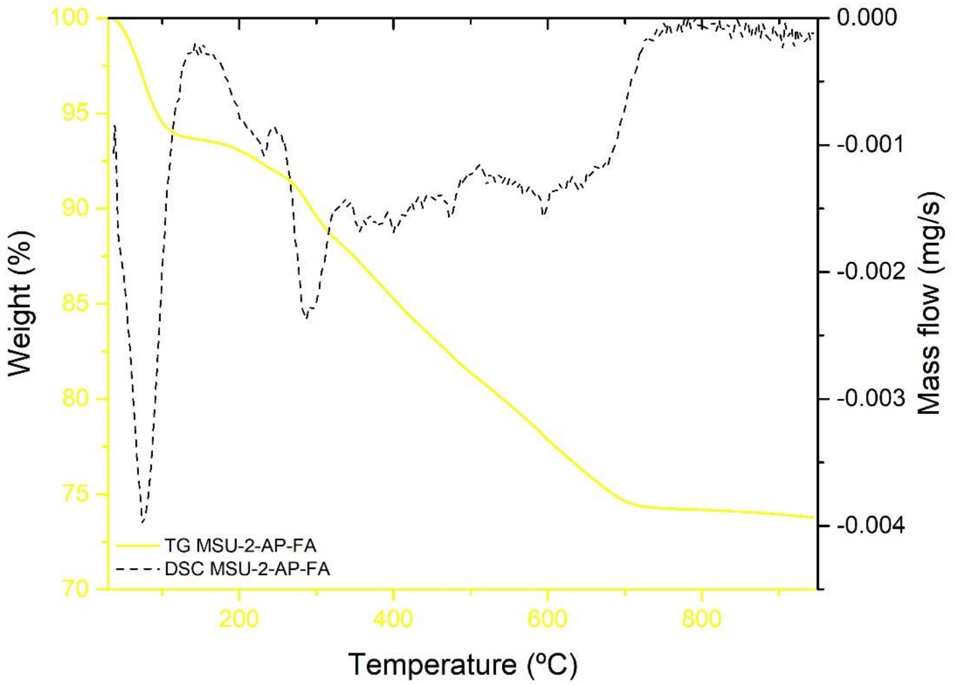

Figure S7. TG-DSC of MSU-2-AP-FA.

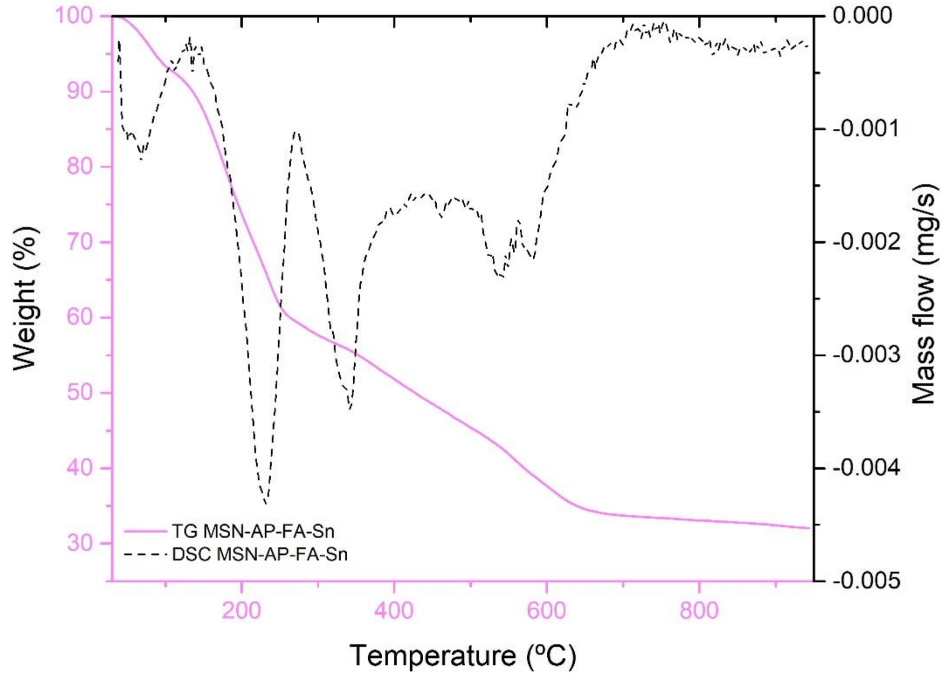

Figure S8. TG-DSC of MSN-AP-FA-Sn.

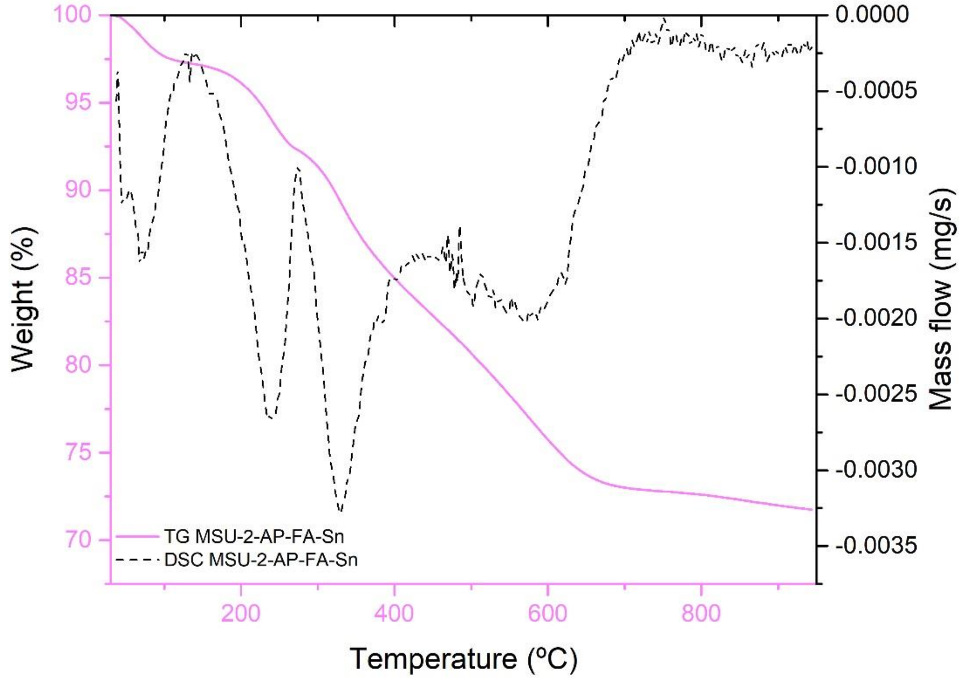

Figure S9. TG-DSC of MSU-2-AP-FA-Sn.

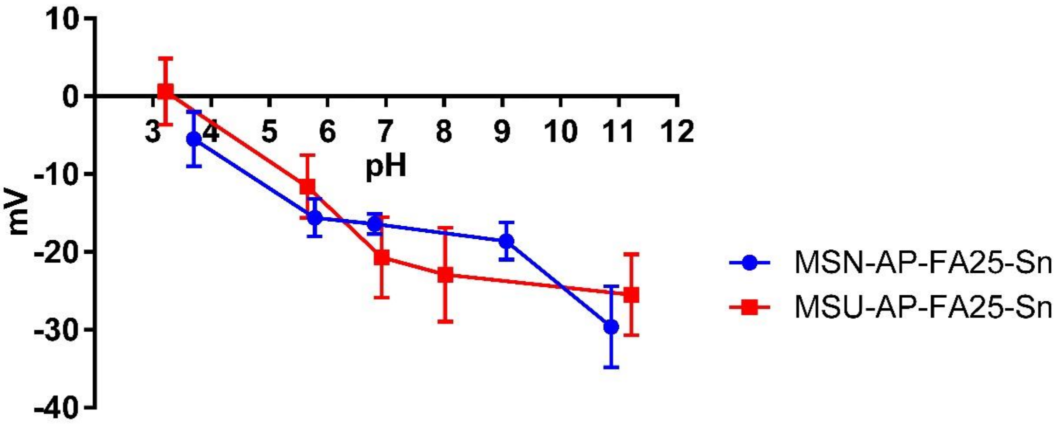

Figure S10. Z-potential measurements between pH 3 and pH 12 for MSN-AP-FA25-Sn and MSU-2-AP-FA25-Sn

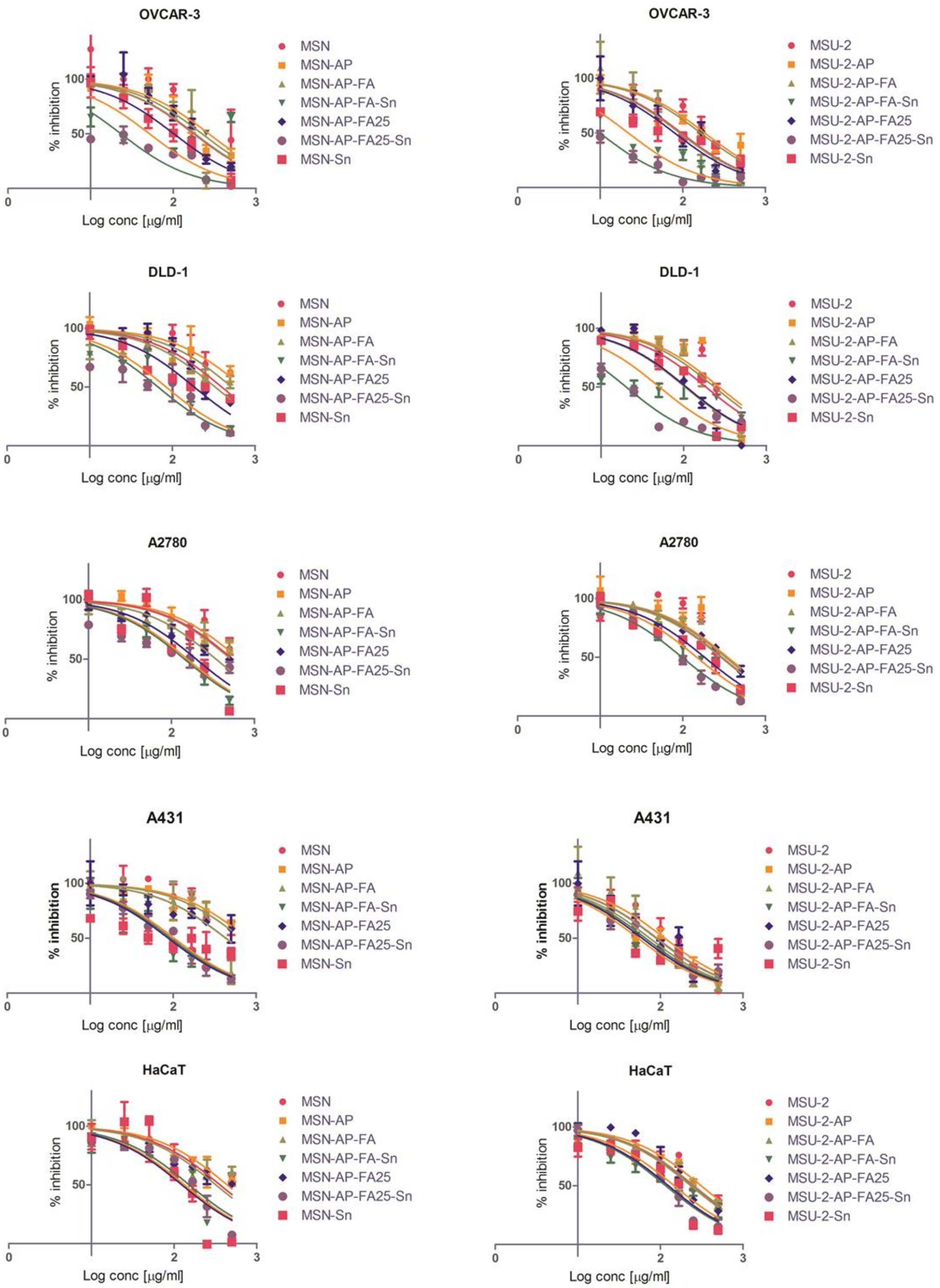

Figure S11. Nonlinear dose-response relationship between the concentration *vs* the cell growth inhibition following the 24-hours treatment with MSU-2 and MSN materials *in vitro*.

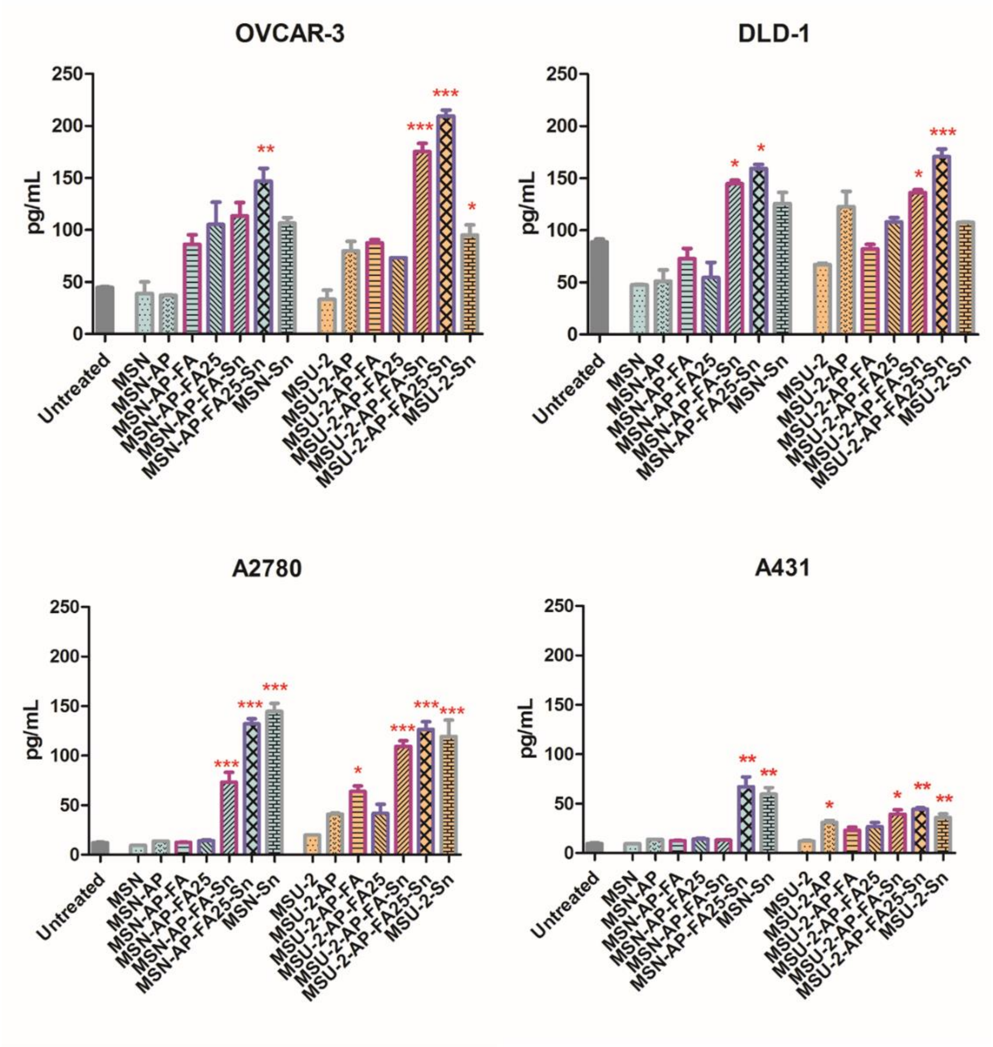

Figure S12. Concentration of FOLR1 secreted by tumour cells subjected to treatment with mesoporous compounds. In the upper row the folate receptor-positive DLD-1 and OVCAR-3 cells, in the lower row are the A2780 and A431 cells, with low folate receptor expression. The statistically significant changes in FOLR1 level *vs* the untreated reference are evidenced by starring: * represents $p<0.05$, ** $p<0.01$ while *** $p<0.001$. The columns with magenta border represents the materials functionalized with 10%FA, the blue border represents the materials functionalized with 25% FA.
